# Emergence and spread of multirecombinant enterovirus A71 lineages C1.3/RF5 and C1.3/RF6 associated with neurological diseases in Europe, 2016–2022, ENPEN multicentre study

**DOI:** 10.1093/ve/veag051

**Published:** 2026-08-08

**Authors:** Belele Siméon Bakyono, Audrey Mirand, Nadia Neuner-Jehle, Cristina Andrés, Jorgina Vila, Andrés Antón, Isabelle Schuffenecker, Marion Jeannoël, Laurence Josset, Elisabeth Toverud Landaas, Suzanne Dudman, Mária Takács, Erika Bujáki, Ágnes Farkas, Laura Pellegrinelli, Elena Pariani, Sandro Binda, Sara Uceda Renteria, Jacky Flipse, Caroline M A Swanink, Jaco J Verweij, Jean-Luc Murk, Karen Couderé, Magdalena Wieczorek, Kacper Toczylowski, Artur Sulik, Maria Milagrosa Montes Ros, Luis Piñeiro, Eider Oñate Vergara, Iñigo Ansa Isasa, Jan Richter, Christina Christodoulou, Ondřej Cinek, Kateřina Chudá, Petr Hubáček, Antonio Piralla, Federica A M Giardina, Paula Palminha, Raquel Neves, Carlos Ribeiro, Maja M Lunar, Mario Poljak, Anne J Jääskeläinen, Baiba Niedre-Otomere, Cécile Henquell, Emma B Hodcroft, Sofie E Midgley, Natasa Berginc, Thea K Fischer, Heli Harvala, Kimberley Benschop, Jean-Luc Bailly, Maxime Bisseux

**Affiliations:** Laboratoire Microorganismes, Génome et Environnement, Université Clermont Auvergne, UMR CNRS 6023, 28 Place Henri Dunant, 63000 Clermont-Ferrand, Auvergne, France; Laboratoire Microorganismes, Génome et Environnement, Université Clermont Auvergne, UMR CNRS 6023, 28 Place Henri Dunant, 63000 Clermont-Ferrand, Auvergne, France; Laboratoire de Virologie, Centre National de Reference des Entérovirus et Parechovirus, Centre Hospitalier Universitaire de Clermont-Ferrand, 58 rue Montalembert, 63000 Clermont-Ferrand, Auvergne, France; Swiss Tropical and Public Health Institute, Kreuzstrasse 2, 4123 Allschwil, Switzerland, University of Basel, Petersplatz 1, Basel, Switzerland; Swiss Institute of Bioinformatics, 1015, Lausanne, Switzerland; Respiratory Viruses Unit, Microbiology Department, Vall d’Hebron Hospital Universitari, Vall d’Hebron Institut de Recerca, Vall d’Hebron Barcelona Hospital Campus, Passeig Vall d'Hebron 119-129, 08035 Barcelona, Catalonia, Spain; Pediatric Hospitalization Unit, Hospital Infantil i de la Dona Vall d’Hebron, Vall d’Hebron Barcelona Hospital Campus, Passeig Vall d'Hebron 119-129, 08035 Barcelona, Catalonia, Spain; Infection and Immunity in Pediatric Patients Research Group, Vall d’Hebron Institut de Recerca, Passeig Vall d'Hebron 119-129, 08035 Barcelona, Catalonia, Spain; Respiratory Viruses Unit, Microbiology Department, Vall d’Hebron Hospital Universitari, Vall d’Hebron Institut de Recerca, Vall d’Hebron Barcelona Hospital Campus, Passeig Vall d'Hebron 119-129, 08035 Barcelona, Catalonia, Spain; Biomedical Research Networking Center in Infectious Diseases, Instituto de Salud Carlos III, Av. Monforte de Lemos, 3-5. Pabellón 11. Planta 0 28029, Madrid, Spain; Centre National de Référence des Enterovirus et Parechovirus—Laboratoire Associé, Institut des Agents Infectieux, Hospices Civils de Lyon, 3 Quai des Célestins, 69002 Lyon, France; Centre National de Référence des Enterovirus et Parechovirus—Laboratoire Associé, Institut des Agents Infectieux, Hospices Civils de Lyon, 3 Quai des Célestins, 69002 Lyon, France; Centre National de Référence des Enterovirus et Parechovirus—Laboratoire Associé, Institut des Agents Infectieux, Hospices Civils de Lyon, 3 Quai des Célestins, 69002 Lyon, France; Plateforme de Séquençage des Pathogènes, Hospices Civils de Lyon, 3 Quai des Célestins, 69002 Lyon, France; Department of Microbiology, Oslo University Hospital; Institute of Clinical Medicine, University of Oslo, Postboks 4950 Nydalen, OUS HF, Rikshospitalet, 0424, Oslo, Norway; Department of Microbiology, Oslo University Hospital; Institute of Clinical Medicine, University of Oslo, Postboks 4950 Nydalen, OUS HF, Rikshospitalet, 0424, Oslo, Norway; Department of Virology, National Centre for Public Health and Pharmacy, Albert Flórián út 2-6, 1097 Budapest, Hungary; Semmelweis University, Institute of Medical Microbiology, Nagyvárad tér 4. X. emelet, 1089, Budapest, Hungary; Medicines and Healthcare Products Regulatory Agency, Blanche Lane, South Mimms, Hertfordshire, EN6 3QG, United Kingdom; Department of Virology, National Centre for Public Health and Pharmacy, Albert Flórián út 2-6, 1097 Budapest, Hungary; Department of Biomedical Sciences for Health, University of Milan, Via Mangiagalli, 3120133, Milan, Italy; Department of Biomedical Sciences for Health, University of Milan, Via Mangiagalli, 3120133, Milan, Italy; Department of Biomedical Sciences for Health, University of Milan, Via Mangiagalli, 3120133, Milan, Italy; Microbiology and Virology Unit, Foundation Istituti di Ricovero e Cura a Carattere Scientifico (IRCCS) Ca’ Granda Ospedale Maggiore Policlinico, Via Francesco Sforza 28, Milan, Italy; Department of Medical Microbiology and Immunology, Rijnstate Hospital, Wagnerlaan 55, 6815 AD, Arnhem, The Netherlands; Laboratory for Medical Microbiology and Immunology, Dicoon, Industrieweg 5, 6662 PA, Elst, The Netherlands; Department of Medical Microbiology and Immunology, Rijnstate Hospital, Wagnerlaan 55, 6815 AD, Arnhem, The Netherlands; Microvida Laboratory of Medical Microbiology and Immunology, Elisabeth-Tweesteden Hospital, PO Box 90151, 5000, LC, Tilburg, The Netherlands; Microvida Laboratory of Medical Microbiology and Immunology, Elisabeth-Tweesteden Hospital, PO Box 90151, 5000, LC, Tilburg, The Netherlands; Microvida Laboratory of Medical Microbiology and Immunology, Elisabeth-Tweesteden Hospital, PO Box 90151, 5000, LC, Tilburg, The Netherlands; Department of Virology, National Institute of Public Health NIH (National Institute of Hygiene) —National Research Institute, 24 Chocimska Street, 00791 Warsaw, Poland; Department of Pediatric Infectious Diseases, Medical University of Bialystok, ul. Waszyngtona 1715-274 Białystok, Poland; Department of Pediatric Infectious Diseases, Medical University of Bialystok, ul. Waszyngtona 1715-274 Białystok, Poland; Microbiology Department, Donostia University Hospital, Biogipuzkoa Health Research Institute, Begiristain Doktorearen Pasealekua, 109, San Sebastián, Gipuzkoa, Spain; Microbiology Department, Donostia University Hospital, Biogipuzkoa Health Research Institute, Begiristain Doktorearen Pasealekua, 109, San Sebastián, Gipuzkoa, Spain; Paedriatric Department, Donostia University Hospital, Biogipuzkoa Health Research Institute, Begiristain Doktorearen Pasealekua, 109, San Sebastián, Gipuzkoa, Spain; Microbiology Department, Donostia University Hospital, Biogipuzkoa Health Research Institute, Begiristain Doktorearen Pasealekua, 109, San Sebastián, Gipuzkoa, Spain; Department of Molecular Virology, Cyprus Institute of Neurology and Genetics, Iroon Avenue 6, Egkomi, 2371 Nicosia, Cyprus; Department of Molecular Virology, Cyprus Institute of Neurology and Genetics, Iroon Avenue 6, Egkomi, 2371 Nicosia, Cyprus; Second Faculty of Medicine, Charles University in Prague, University Hospital Motol and Homolka, V Úvalu 84, 150 06, Prague 5, Czech Republic; Second Faculty of Medicine, Charles University in Prague, University Hospital Motol and Homolka, V Úvalu 84, 150 06, Prague 5, Czech Republic; Second Faculty of Medicine, Charles University in Prague, University Hospital Motol and Homolka, V Úvalu 84, 150 06, Prague 5, Czech Republic; Microbiology and Virology Department, Fondazione IRCCS Policlinico San Matteo, Viale Camillo Golgi 19 - 27100, Pavia, Lombardia, Italy; Department of Clinical, Surgical, Diagnostic and Pediatric Sciences, University of Pavia, Campus della Salute, presso Policlinico San Matteo, Viale Golgi 19, 27100 Pavia, Italy; National Reference Laboratory for Vaccine Preventable Diseases, Department of Infectious Diseases, Instituto Nacional de Saúde Dr. Ricardo Jorge (INSA), Av. Padre Cruz, 1649-016, Lisbon, Portugal; National Reference Laboratory for Vaccine Preventable Diseases, Department of Infectious Diseases, Instituto Nacional de Saúde Dr. Ricardo Jorge (INSA), Av. Padre Cruz, 1649-016, Lisbon, Portugal; National Reference Laboratory for Vaccine Preventable Diseases, Department of Infectious Diseases, Instituto Nacional de Saúde Dr. Ricardo Jorge (INSA), Av. Padre Cruz, 1649-016, Lisbon, Portugal; Institute of Microbiology and Immunology, Faculty of Medicine, University of Ljubljana, Zaloška 4, 1000 Ljubljana, Slovenia; Institute of Microbiology and Immunology, Faculty of Medicine, University of Ljubljana, Zaloška 4, 1000 Ljubljana, Slovenia; Diagnostic Center, Virology and Immunology, University of Helsinki and Helsinki University Hospital (HUS), Stenbäckinkatu 9, Helsinki, Finland; National Microbiology Reference Laboratory of Latvia, Laboratory ‘Latvian Centre of Infectious Diseases’, Laboratory Service, Riga East University Hospital, Linezera Street 3, LV-1006, Riga, Latvia; Laboratoire Microorganismes, Génome et Environnement, Université Clermont Auvergne, UMR CNRS 6023, 28 Place Henri Dunant, 63000 Clermont-Ferrand, Auvergne, France; Laboratoire de Virologie, Centre National de Reference des Entérovirus et Parechovirus, Centre Hospitalier Universitaire de Clermont-Ferrand, 58 rue Montalembert, 63000 Clermont-Ferrand, Auvergne, France; Swiss Tropical and Public Health Institute, Kreuzstrasse 2, 4123 Allschwil, Switzerland, University of Basel, Petersplatz 1, Basel, Switzerland; Swiss Institute of Bioinformatics, 1015, Lausanne, Switzerland; Department of Virology and Mircrobiology Preparedness, Statens Serum Institut, Artillerivej 5, 2300 Kbh. S., Copenhagen, Denmark; National Laboratory of Health, Environment and Food, Laboratory for Public Health Virology, Zaloška cesta 29 | 1000, Ljubljana, Slovenia; Department Clinical Research, Nordsjaellands Hospital, Dyrehavevej 29, 3400, Hilleroed, Denmark; University of Copenhagen, Øster Farimagsgade 5 opg. B1353, Copenhagen, Denmark; Institute of Biomedicine, Faculty of Medicine; Medical Microbiology, Turku University Hospital, Kiinamyllynkatu 10, FI-20520, Turku, Finland; Centre for Infectious Disease Control, Centre for Infectious Disease Research, Diagnostics and Laboratory Surveillance Dutch National Institute for Public Health and the Environment, Antonie van Leeuwenhoeklaan, 93721 MA, Bilthoven, The Netherlands; Laboratoire Microorganismes, Génome et Environnement, Université Clermont Auvergne, UMR CNRS 6023, 28 Place Henri Dunant, 63000 Clermont-Ferrand, Auvergne, France; Laboratoire Microorganismes, Génome et Environnement, Université Clermont Auvergne, UMR CNRS 6023, 28 Place Henri Dunant, 63000 Clermont-Ferrand, Auvergne, France; Laboratoire de Virologie, Centre National de Reference des Entérovirus et Parechovirus, Centre Hospitalier Universitaire de Clermont-Ferrand, 58 rue Montalembert, 63000 Clermont-Ferrand, Auvergne, France

**Keywords:** enterovirus A71, clinical manifestations, transmission, evolutionary dynamics, emerging lineages

## Abstract

Since its re-emergence in Europe in 2015, enterovirus A71 (EV-A71) has been associated with sporadic cases of severe infections and outbreaks. Currently, there is no treatment but a few Asian countries have implemented vaccination programmes. The aim of our study was to characterize the EV-A71 cases in Europe and track the evolution of the virus between 2016 and 2022.

For this retrospective study, we compiled the data of confirmed EV-A71 infections collected by 19 institutes participating in the European Non-Polio Enterovirus Network between 2016 and 2022. We also sequenced whole EV-A71 genomes to follow the viral evolutionary history of the virus and its molecular changes.

Between 1 January 2016 and 31 December 2022, 627 EV-A71 cases were reported in 14 European countries. Most cases (59.3%) occurred in children under two years of age. Of the 537 subjects for whom clinical information was available, 52.7% were diagnosed with neurological signs, and 10.6% presented with acute flaccid paralysis or acute flaccid myelitis. EV-A71 infections were linked to two subclades, C1.2 and C1.3, within the C1 subgenogroup. Two marked epidemic waves, in 2016 and 2019, were characterized by the predominance of C1.3 viruses. Comparisons of 153 whole genomes revealed nine recombinant forms (RFs), each arising from multiple recombination events affecting different genomic regions. Only C1.3/RF5 and C1.3/RF6 lineages achieved widespread geographic dissemination initially in Europe and subsequently in Asia and the Americas, and long-term circulation. Phylodynamic analyses indicated that C1.3 viruses emerged around 2013–2014, displacing earlier C1.1 viruses, and the effective population size closely parallels the timing of clinical cases. Molecular analyses of the capsid revealed several amino acid changes, few of which occurred within known antigenic sites.

These findings document the role of the C1.3/RF5 and C1.3/RF6 multi-recombinant lineages of EV-A71 as frequent causes of neurological disease in young children in Europe. The study highlights the need for sustained genome-informed surveillance to anticipate the emergence and spread of novel epidemic lineages, and to inform the evaluation of future EV-A71 vaccination strategies.

## 1. Introduction

Enterovirus A71 (EV-A71) is a common cause of recurring epidemics worldwide (ECDC 2016, Puenpa et al. 2019). While most EV-A71 infections are considered subclinical, this virus can cause mild and self-limiting hand, foot, and mouth disease (HFMD), which primarily affects children under the age of five years during large outbreaks (Solomon et al. 2010). HFMD has been reported to progress in some cases to severe neurological infections in young children and sometimes fatal diseases such as cardiopulmonary oedema (Corvest et al. 2017, Luciani et al. 2021). Since the late 1990s, there have been large outbreaks of HFMD associated with EV-A71 across the Asia-Pacific region, including in Malaysia, Singapore, Taiwan, China, Vietnam, and Cambodia (Huang et al. 2024). These outbreaks have led to substantial morbidity and mortality, and in a few Asian countries have prompted the development and deployment of inactivated EV-A71 vaccines (Liu et al. 2025). In Europe, EV-A71 circulation has been documented for more than two decades through national enterovirus surveillance systems, with both sporadic cases and localized outbreaks reported in several countries (Bible et al. 2008, Mirand et al. 2010, Badran et al. 2011, Cabrerizo et al. 2014, Fischer et al. 2014, Hassel et al. 2015). Recent episodes of increased EV-A71 circulation associated with neurological disease in children, such as the 2016 outbreak in Spain and subsequent events in other European countries, have been linked to the emergence or re-emergence of specific lineages (Böttcher et al. 2016, Midgley et al. 2017, González-Sanz et al. 2019, Ngangas et al. 2019) occasionally associated with severe presentations (Karrasch et al. 2016, Casas-Alba et al. 2017, Luciani et al. 2021). These epidemiological events have consequently driven a growing need for broad research into the evolutionary and spatiotemporal dynamics of EV-A71 in Europe, to better understand the origins, spread, and genetic diversity driving these outbreaks, and associated disease severity. Most studies on the molecular epidemiology of EV-A71 rely on partial VP1 sequences and typically focus on single countries or limited time windows, providing only a fragmented view of continent-wide patterns of EV-A71 evolution, recombination, and geographic spread. This hampers comprehensive risk assessment, including evaluation of the potential impact of vaccine introduction and the likelihood of further emergence of neurovirulent variants.

EV-A71 belongs to the *Enterovirus alphacoxsackie* (ex EV-A) species and is genetically diverse. Strains circulating worldwide are classified into genogroups A–H (Brown et al. 1999, Bessaud et al. 2014, Majumdar et al. 2018). The two main genogroups B and C are further subdivided into multiple subgenogroups, B1–B5 and C1–C5. Like other EV types, EV-A71 evolves through the combined effects of high substitution rates and recombination within the non-structural P2 and P3 regions (McWilliam Leitch et al. 2012, Lee et al. 2018). Consequently, circulating viral strains often display mosaic genomes, in which structural and non-structural regions have distinct evolutionary histories because non-structural genomic regions are shared by different EV types (Nikolaidis et al. 2019). Detailed whole-genome analyzed are therefore required to identify recombinant forms (RFs), quantify their contribution to local and regional epidemics, and determine whether specific RFs or capsid lineages are preferentially associated with severe neurological disease.

To address these gaps in knowledge in the European setting, the European Non-Polio Enterovirus Network (ENPEN) coordinated a multi-country effort to assemble standardized clinical, epidemiological, and genomic data on EV-A71 infections reported between 2016 and 2022. Participating institutes reported clinical and epidemiological metadata for laboratory-confirmed EV-A71 cases together with partial and complete genome sequences, enabling a continent-wide analysis of recent EV-A71 molecular epidemiology. In the present study, near-complete genomes and VP1 sequences were analysed to (i) describe the clinical and demographic characteristics of EV-A71 cases, (ii) delineate the genetic structure and recombinant diversity of circulating C1 lineages, and (iii) reconstruct the evolutionary history and geographic spread of emergent epidemic viruses. This integrative framework aims to clarify the drivers of recent EV-A71 activity in Europe and to inform future surveillance needs, risk assessment and control strategies, including potential vaccine implementation.

## 2. Materials and methods

### 2.1. Sample collection and data sharing

A call for sample submissions was disseminated through ENPEN to participate in this study on a voluntary basis. In total, 19 institutes from 14 European countries (Cyprus, the Czech Republic, Denmark, Finland, France, Hungary, Italy, Latvia, the Netherlands, Norway, Poland, Portugal, Slovenia, and Spain) contributed to the study (Table 1). Each participant systematically reported each EV-A71 infection case identified through routine molecular diagnostics. Virological testing was performed according to the specific procedures in use at each participating laboratory. Contributing institutes were requested to provide the following for each EV-A71 case: anonymized clinical metadata, the longest genomic fragments obtained during viral typing, and, when available, residual specimen material suitable for complete genome sequencing.

**Table 1:** EV-A71 sequence data analysed for the EV-A71 cases reported between 1 January 2016 and 31 December 2022.

Participant^#^	Country^a^	Location of participating institute	Partial VP1^b^	Complete VP1^b^	VP2^b^	P1	Complete (near complete) genome	Total
**1**	**Cyprus**	Nicosia	4	/	/	/	2^**c**^	**6**
**2**	**Czech Republic**	Olomouc	2	/	/	/	4^**c**^	**6**
**3**	**Denmark**	Copenhagen	20	/	31	2	5	**58**
**4**	**Finland**	Helsinki	6	/	/	/	/	**6**
**5**	**France**	Clermont-Ferrand	89	2	/	/	98^**c**^	**189**
**6**	**France**	Lyon	188	/	/	/	/	**188**
**7**	**Hungary**	Budapest	3	/	/	/	/	**3**
**8**	**Italy**	Milan	6	/	/	/	/	**6**
**9**	**Italy**	Pavia	2	/	/	/	/	**2**
**10**	**Latvia**	Riga	/	/	/	/	5	**5**
**11**	**Netherlands**	Rijnstate	7	/	/	/	6	**13**
**12**	**Netherlands**	Tilburg	9	/	/	/	/	**9**
**13**	**Norway**	Oslo	21	/	/	/	/	**21**
**14**	**Poland**	Bialystok	/	/	/	/	5^**c**^	**5**
**15**	**Portugal**	Oporto/Lisbon	6	/	/	/	4	**10**
**16**	**Slovenia**	Ljubljana	5	/	/	/	2^**c**^	**7**
**17**	**Spain**	Barcelona	21	13	/	/	22^**c**^	**56**
**18**	**Spain**	San Sebastian	6	/	/	/	/	**6**
			**395** ^**d**^	**15**	**31**	**2**	**153**	**596**

^#^The institutes participating in the study were assigned numbers. ^a^A second laboratory at the University of Ljubljana (Slovenia) detected no EV-A71-positive case among 130 EV-positive patients tested.

^b^Sequence data obtained through molecular typing performed by participants to the study.

^c^Sequencing performed by participating laboratory # 5.

^d^Sixty sequences of low quality were not used in the phylogenetic analyses.

Data and strain exchanges were governed by formal bilateral transfer agreements between each institution and the coordinating laboratory. All data were anonymized prior to transfer in accordance with national and international ethical regulations.

### 2.2. Sequence data and sequencing of complete enterovirus A71 genomes

A viral nucleotide sequence was obtained for 596 of the 627 EV-A71 cases reported by ENPEN participants and were distributed as shown in Table 1. Thirty-one VP2 sequences and 60 low-quality VP1 sequences—i.e. those lacking full coverage of the minimal region required for strain comparison (positions 2610–2850 in reference strain BrCr, accession number U22521.1)—were excluded from our analyses. The 153 complete or nearly complete EV-A71 genomes analysed in this study were determined in four different participating institutes. The genomes from infection cases reported in Denmark (*n =* 5) were previously sequenced and described (Midgley et al. 2017). Those from the Netherlands (*n =* 6) and Latvia (*n =* 5) were sequenced using the same method. The remaining 137 EV-A71 genomes were sequenced at institute #5 from anonymized clinical specimens or nucleic acids provided by six participants: institute #1 (*n =* 2 samples), #2 (*n =* 4), #5 (*n =* 98), #14 (*n =* 5), #16 (*n =* 2), and #17 (*n =* 22) from 255 clinical specimens. Viral genomes were determined with two sequencing platforms: 30 sequences were obtained with the MinION Mk1C platform (Oxford Nanopore Technologies) and 107 sequences with a NextSeq 500 system (Illumina), following the procedures fully described in the supplementary material.

### 2.3. Construction of analytical enterovirus A71 sequence datasets

The VP1 dataset comprised a total of 505 sequences including 335 high-quality partial VP1 fragments, 15 complete VP1 sequences, and a further 155 complete VP1 sequences extracted from P1 and complete (or near complete) genome sequences (see Table 1). The 505 VP1 sequences were supplemented by 17 complete VP1 reference sequences representing all known genogroups and subgenogroups of EV-A71 as of September 2024 (GenBank), altogether 522 sequences. The second dataset included only C1 sequences and comprised 161 sequences, including the 121 complete C1 genomes listed in Table 1, and 40 additional EV-A71 C1 genomes from GenBank. All sequence alignments were constructed with MAFFT v7 (https://mafft.cbrc.jp/alignment/) and manually curated in BioEdit (Hall 1999).

### 2.4. Similarity analyses of complete enterovirus A71 genomes

Nucleotide identity among complete viral genomes was assessed with SimPlot 3.5.1 (Lole et al. 1999) using a 200 nt sliding window and a 20 nt step size. Percent similarity was plotted across aligned genomic positions. The exact positions of recombination breakpoints were determined using RDP5 (Martin et al. 2021) and are reported relative to the BrCr reference genome (accession number U22521.1).

### 2.5. Maximum likelihood analyses and molecular clock assessment

The IQ-TREE server (http://iqtree.cibiv.univie.ac.at/) was used to reconstruct phylogenetic trees with a maximum likelihood approach. The analysis was performed using the general time reversible (GTR) substitution model, incorporating gamma (Γ)-distributed rate variation (four categories) and estimation of the proportion of invariant sites. Node support was assessed using the ultrafast bootstrap and SH-aLRT (each with 10 000 replicates); values below 85% were not considered. Trees were visualized in FigTree 1.4.4. Molecular clock signal was assessed using TempEst 1.5.3 (Rambaut et al. 2016) by regression of root-to-tip genetic distances against sampling dates. The coefficient of determination (R^2^) was used to quantify temporal signal strength.

### 2.6. Bayesian phylogenetic and phylodynamic analyses

Time-calibrated phylogenies and estimates of population dynamics were generated using BEAST v2.7.4 (Bouckaert et al. 2014), which was run on the supercomputer facility of Université Clermont Auvergne. Analyses employed a relaxed molecular clock model and the GTR + Γ substitution model (four gamma categories with invariant sites) under a Bayesian Skyline coalescent tree prior. A relaxed molecular clock was selected to account for potential rate heterogeneity among viral lineages, as evolutionary rates may vary across branches during virus spread and diversification. Ten independent MCMC chains were run for one billion generations each, sampling every 100 000 steps. Convergence and effective sample sizes (ESS > 3000) were checked in Tracer following 10% burn-in. Maximum clade credibility (MCC) trees were summarized using TreeAnnotator v2.6.6 (Drummond and Rambaut 2007).

### 2.7. Amino acid changes in capsid proteins of enterovirus A71 C1

The Augur pipeline (Hadfield et al. 2018, Huddleston et al. 2021) was used to investigate amino acid substitutions in capsid proteins throughout the evolutionary history of EV-A71 C1. The ENPEN dataset was complemented with EV-A71 sequences, which were retrieved from the GenBank database as of 16 January 2026. The final dataset was aligned and annotated using Nextclade3 (Aksamentov et al. 2021) and the BrCr prototype sequence as a reference (U22521.1). A phylogenetic tree was reconstructed using IQ-TREE (Nguyen et al. 2015) and a time-resolved maximum likelihood tree was generated with TreeTime (Sagulenko et al. 2018). Ancestral sequences at nucleotide and protein levels were reconstructed using Augur and TreeTime, and the changes in nucleotides and amino acids that occurred during evolution of C1 subclades (see the Results section below) were represented across the tree. The complete workflow and the final versions of the generated Nextstrain files are available on GitHub at github.com/enterovirus-phylo/enterovirus_A71.

Three-dimensional models of the capsid pentamer were generated for each EV-A71 C1 subclade using AlphaFold (https://alphafold.ebi.ac.uk/). The reconstructed 3D structures were validated by comparison with the cryo-electron microscopy 3D structure of the closely related C2 subgenogroup native virion (PDB ID: 8E2X; strain 4643; Catching et al. 2023). The structure of subclade C1.2 was reconstructed using the MG367600.1 sequence, while the structure of subclade C1.3 was reconstructed using the LR027531.1 sequence (both from this study). The amino acid changes identified in the modelled structures for each subclade were mapped onto pentameric models and annotated using UCSF Chimera v1.18 (Pettersen et al. 2004).

### 2.8. Data availability

The GenBank accession numbers of the 153 complete EV-A71 genomes analysed in this study are listed in Supplementary Table S1. Multiple sequence alignments, and phylogenetic trees are available from the corresponding authors upon reasonable request.

## 3. Results

### 3.1. Overview of enterovirus A71 clinical cases reported by participating institutes between 2016 and 2022

**Figure 1:**
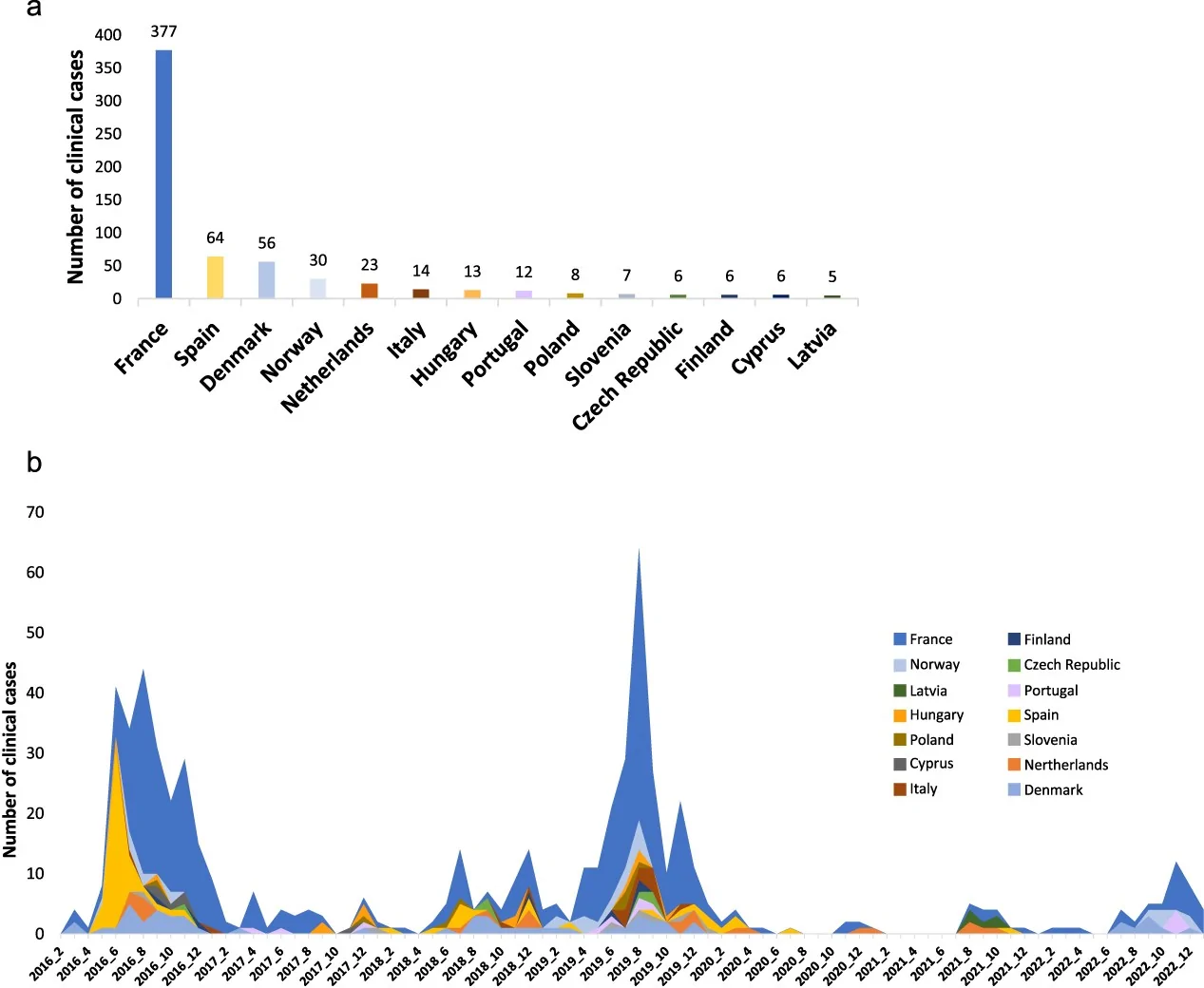
Number and temporal distribution of EV-A71 infection cases reported in 14 European countries. (a) A total of 627 cases of EV-A71 infections were collected between 1 January 2016 and 31 December 2022 by 19 ENPEN participating institutes spread across 14 European countries. (b) The stacked area plot shows the monthly distribution of the EV-A71 infection cases collected for the present study among participating countries.

The complete workflow of data analyses is provided in Supplementary Fig. S1. Between January 2016 and December 2022, the 19 ENPEN participating institutes reported 627 cases of EV-A71 infections across 14 European countries (Fig. 1a). France reported 377 cases, which accounted for 60.1% of all notifications. Spain, Denmark, Norway, and the Netherlands reported 64, 56, 30, and 23 cases, respectively. The remaining nine participating institutes each reported fewer than 20 cases, contributing a total of 77 cases (12.3%). The temporal distribution of notifications showed two distinct epidemic peaks in 2016 and 2019, both occurring between late spring and early autumn (Fig. 1b). These two waves accounted for 65.1% of all reported cases (*n =* 408/627).

**Figure 2:**
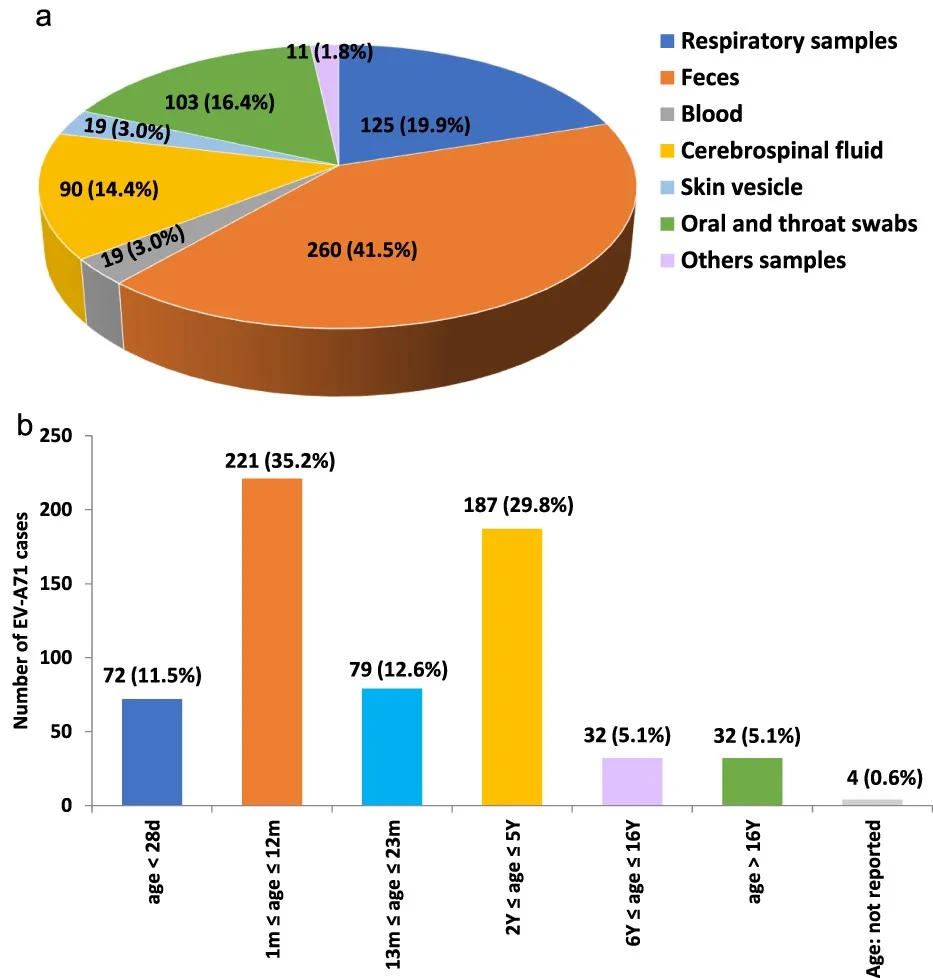
Distribution of biological specimens used for diagnostic testing (a) and patient age (b) among the 627 EV-A71 infection cases reported by the participating ENPEN institutes between 2016 and 2022.

EV-A71 was detected in multiple types of biological specimens (Fig. 2a). Stool specimens accounted for 41.5% of detections (*n =* 260/627), followed by respiratory samples (19.9%, *n =* 125), oral and throat swabs (16.4%, *n =* 103), and CSF (14.3%, *n =* 90). Most cases occurred in children under 2 years of age (59.3%, *n =* 372), followed by those aged 2–5 years (29.8%, *n =* 187) and those older than 5 years (10.2%, *n =* 64) (Fig. 2b). Overall, 71.0% (*n =* 445/627) of cases were identified in hospitalized patients, including 15.7% (*n =* 70/445) in intensive care, and 13.9% (*n =* 87/627) through ambulatory surveillance. Diagnostic context was not documented for 95 of the 627 (15.2%) EV-A71 infection cases.

### 3.2. Clinical signs reported at diagnosis

Diagnostic information was documented for 537/627 (85.6%) patients. Of these 537 patients, 283 (52.7%) had a neurological disease defined by non-mutually exclusive signs, including meningitis, encephalitis, acute flaccid myelitis (AFM) or acute flaccid paralysis (AFP), and other signs (e.g. seizures, headache without meningitis, irritability) (see Table 2). Encephalitis and meningitis were described in 23.1% and 14.9% of patients, respectively. AFM and AFP together were observed in 10.6% (*n =* 57) and other neurological signs in 16.8% (*n =* 90) of patients. AFP/AFM cases were reported in five countries: Czech Republic (*n =* 2), France (*n =* 20), Hungary (*n =* 2), Italy (*n =* 4), and Spain (*n =* 29). Half of the patients with AFP/AFM (49.1%) were younger than two years, 38.6% were aged between two and five years of age, and 12.3% were older than five years. All but six were infected with subgenogroup C1 viruses. The other six AFP/AFM patients had an EV-A71 C2 infection. Extra-neurological signs were also reported in 61.6% of the patients (*n =* 331/537), including HFMD and muco-cutaneous signs (31.3%), respiratory signs (25.1%), gastrointestinal signs (25%) and cardiac involvement (2.6%).

**Table 2:** Clinical characteristics available in EV-A71 case reported among European countries between 2016 and 2022.^a^

Clinical characteristics available at diagnosis^b^	Cyprus	Czech Republic	Finland	France	Hungary	Italy	Latvia	Netherlands	Norway	Poland	Portugal	Slovenia	Spain	Total case number
**Number of patients with clinical information available**	**6**	**6**	**4**	**354**	**13**	**14**	**1**	**23**	**27**	**8**	**10**	**7**	**64**	**537**
**Meningitis**	3	1	/	55	3	2	1	5	2	7	/	/	1	**80 (14.9%)**
**Encephalitis**	3	/	/	63	5	3	/	/	6	1	5	/	38	**124 (23.1%)**
**AFM or AFP**	/	2	/	20	2	4	/	/	/	/	/	/	29	**57 (10.6%)**
**Other neurological signs** ^**c**^	/	2	/	54	2	/	1	1	4	/	5	/	21	**90 (16.8%)**
**HFMD and muco-cutaneous signs**	/	/	2	121	3	/	/	6	8	1	/	3	24	**168 (31.3%)**
**Intestinal or gastric signs**	/	/	3	90	1	/	/	9	1	2	/	/	28	**134 (25%)**
**Respiratory signs**	/	/	1	79	2	10	/	5	5	/	/	5	28	**135 (25.1%)**
**Cardiac signs**	/	/	1	9	/	/	/	/	2	/	/	/	2	**14 (2.6%)**
**Other signs** ^**d**^	/	1	/	36	/	/	/	5	/	/	/	1	/	**43 (8.0%)**

^a^Clinical information was not available for patients from Denmark.

^b^Clinical signs are not mutually exclusive, implying that the sum of clinical signs is greater than the number of patients.

^c^Convulsions, irritability, headache in the absence of meningitis.

^d^Patients hospitalized with isolated febrile illness.

The values in bold are the total number of patient or symptoms and the row and column headers.

### 3.3. Virus identification

**Figure 3:**
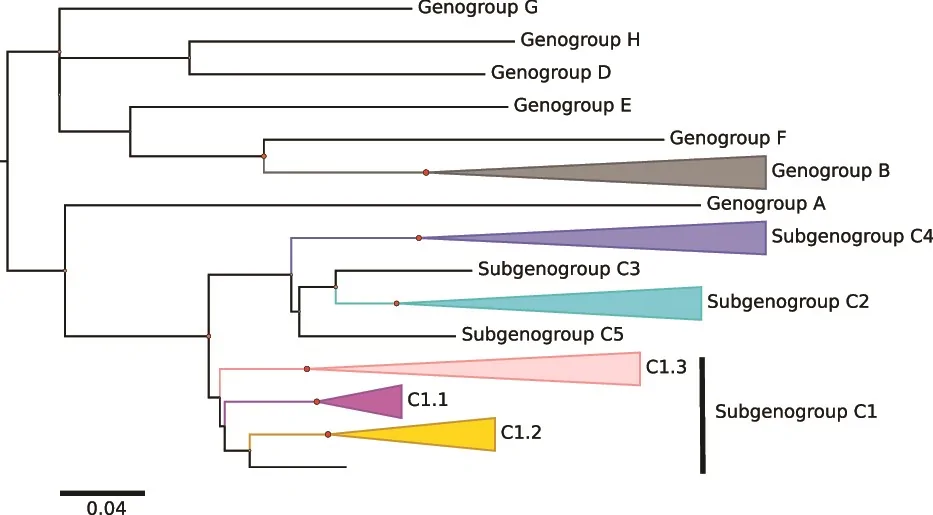
Phylogenetic clustering of EV-A71 VP1 sequences and assignment to extant genogroups. The phylogenetic tree was reconstructed using a maximum likelihood method from 522 VP1 sequences, including the 505 sequences available from the participating institutes (see Table 1) and 17 reference sequences from GenBank. Sequences from the present study were collapsed into coloured clusters (C4, C2, C1.1, C1.2, and C1.3) for greater clarity. Nodes with high SH-like test support are highlighted by circles. Only one reference sequence was used for genogroups A, D, E, F, G, and H, and subgenogroups C1, C3, and C5. The extend tree is shown in Supplementary Fig. S1.

Results for genogroup identification and/or partial VP1 or VP2 typing sequences were available for 596 out of 627 (95.0%) EV-A71 cases from the 19 participating institutes. Based on these data, EV-A71 subgenogroup C1 was the predominant lineage, detected in 85.9% of cases (*n =* 512/596). Subgenogroup C2 accounted for 13.8% (*n =* 82/596), while subgenogroup C4 was detected only sporadically (<1%, *n =* 2/596). We conducted a phylogenetic analysis of 505 high-quality VP1 sequences to identify the virus strains more precisely (Fig. 3). The analysis showed that 438 sequences (86.7%) belonged to subgenogroup C1 and were found in all 14 participating countries (Table 3). A further 65 sequences (12.9%) were assigned to subgenogroup C2 and were found in six countries, and two subgenogroup C4 sequences (0.4%) were found in two countries. The C1 sequences, which were highly diverse, were distributed into three distinct subclades designated C1.1 (*n =* 9/438 sequences), C1.2 (*n =* 11/438), and C1.3 (*n =* 418/438). The detailed phylogenetic tree and the distribution of sequences across countries are given in the supplementary material (Supplementary Fig. S2).

**Table 3:** Distribution of EV-A71 among subclades and countries based on VP1 sequences analysed in this study.^a^

Country	Subclade C1.1	Subclade C1.2	Subclade C1.3	Subclade C2	Subclade C4	All subclades C1 (total)	Total sequences
**Cyprus**	3		3			6	**6**
**Czech Republic**	1	2	3			6	**6**
**Denmark**	2	1	19		1	22	**23**
**Finland**	1		5			6	**6**
**France**	2	8	268	55		278	**333**
**Hungary**			3			3	**3**
**Italy**			5	3		5	**8**
**Latvia**			5			5	**5**
**Netherlands**			21	1		21	**22**
**Norway**			20			20	**20**
**Poland**			3	2		3	**5**
**Portugal**			7	2		7	**9**
**Slovenia**			6		1	6	**7**
**Spain**			50	2		50	**52**
**Total**	**9 (1.8%)**	**11 (2.2%)**	**418 (82.8%)**	**65 (12.9%)**	**2 (0.4%)**	**438 (86.7%)**	**505**

^a^The table indicates the number of EV-A71 sequences assigned to phylogenetic subclades in each participating country.

The values in bold are the total sums per column and row, and the row and column headers.

### 3.4. Detection and characterization of enterovirus A71 recombinant forms

**Figure 4:**
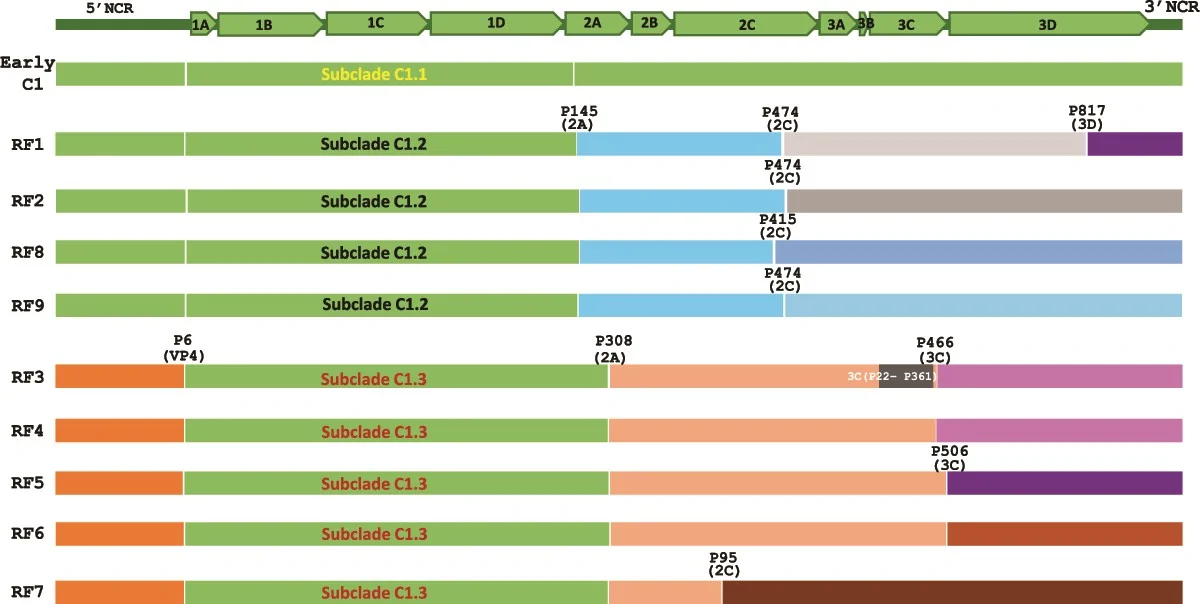
Schematic representation of nine RFs identified among EV-A71 genomes analysed in this study. Of the 153 complete or near-complete genomes investigated, nine distinct RFs were identified based on similarity profiles generated in SimPlot. For each RF, recombinant regions are annotated with distinct colours relative to a C1.1 reference genome (i.e. EV-A71 C1 circulating before 2015). Identical colour across RFs indicates shared genomic segments, while differing colours represent genomic regions of independent recombinant origin. The most probable positions of recombination breakpoints determined using RDP5 were represented: they are reported relative to the BrCr reference genome (accession number U22521.1) and annotated according to their locations within the corresponding genes. The RF3 genome was reported before the study period, in 2014 in Denmark (Midgley et al. 2017) but was included owing to its genetic relationship with other RFs (see also Fig. 5a).

Genetic diversity was investigated at the scale of the whole viral genomes by a comprehensive assessment of nucleotide similarity between 153 complete or near complete EV-A71 genomes, including 5 already available sequences and 148 sequences determined for this study (Supplementary Fig. S3; Supplementary Table S1). The data showed nine discrete recombinant lineages, herein designated RF1 to RF9, belonging to either subclades C1.2 or C1.3. The locations of recombination breakpoints were assessed with different statistical methods to determine how the nine RFs diverged from each other and the early C1.1 genomes (Table 4). The overall structure of RFs and probable breakpoints of recombination are schematized in Fig. 4. The recombination breakpoints were located with confidence in three principal genomic loci: 5′NCR, 2A gene sequence, and 2C gene sequence. A complete analysis of sequence changes between EV-A71 RFs is provided in the supplementary material (Supplementary Fig. S3). Two RFs were the most frequent among the EV-A71 genomes analysed in this study: 92 genomes from ten countries were assigned to RF5 and 32 genomes from five countries to RF6. The other RFs were detected only sporadically: RF9 was identified twice, and RF1–RF4, RF7, and RF8 were each observed once. Of these sporadic lineages, RF2, RF4, RF7, and RF8 were found in France, RF1 and RF3 in Denmark and RF9 in the Czech Republic.

**Table 4:** Localisation of recombination sites and most probable recombination breakpoint intervals.

RF	Probable breakpoint location (gene-position)	Probable interval of breakpoint location with 95% confidence	RDP	GENECONV	Bootscan	MaxChi	Chimera	SiScan	3Seq
**RF1**	2A-145	2A-124—2A-196	1.4.10^−28^	6.2.10^−27^	4.5.10^−32^	8.3.10^−17^	5.9.10^−09^	1.9.10^−33^	2.6.10^−21^
	2C-474	2C-440—2C-468	1.7.10^−10^	7.9.10^−11^	5.6.10^−14^	2.5.10^−10^	3.4.10^−07^	2.0.10^−28^	1.7.10^−03^
	3D-817	3D-783—3D-817	8.0.10^−35^	3.7.10^−33^	1.5.10^−35^	6.1.10^−12^	1.4.10^−09^	5.9.10^−25^	6.7.10^−09^
**RF2**	2A-145	2A-124–2A-196	1.4.10^−28^	6.2.10^−27^	4.5.10^−32^	8.3.10^−17^	5.9.10^−09^	1.9.10^−33^	2.6.10^−21^
	2C-474	2C-440–2C-468	1.7.10^−10^	7.9.10^−11^	5.6.10^−14^	2.5.10^−10^	3.4.10^−07^	2.0.10^−28^	1.7.10^−03^
**RF8**	2A-145	2A-124–2A-196	1.4.10^−28^	6.2.10^−27^	4.5.10^−32^	8.3.10^−17^	5.9.10^−09^	1.9.10^−33^	2.6.10^−21^
	2C-415	2C-389–2C-474	1.6.10^−05^	1.8. 10^−08^	9.10^−12^	3.7.10^−13^	8.6.10^−10^	1.1.10^−45^	1.8.10^−12^
**RF9**	2A-145	2A-124–2A-196	1.4.10^−28^	6.2.10^−27^	4.5.10^−32^	8.3.10^−17^	5.9.10^−09^	1.9.10^−33^	2.6.10^−21^
	2C-474	2C-440–2C-468	1.7.10^−10^	7.9.10^−11^	5.6.10^−14^	2.5.10^−10^	3.4.10^−07^	2.0.10^−28^	1.7.10^−03^
**RF3** ^**a**^	VP4–175	5NCR-738—VP4–195	6.2.10^−12^	7.9.10^−15^	5.5.10^−12^	4.0.10^−06^	3.0.10^−07^	2.4.10^−23^	/^**b**^
	2A-308	2A-248–2A-326	5.8.10^−20^	7.6.10^−18^	2.5.10^−20^	3.6.10^−08^	6.8.10^−05^	5.6.10^−54^	8.9.10^−08^
	3C-466	3C-361–3C-517	5.8.10^−20^	7.6.10^−18^	2.5.10^−20^	1.7.10^−18^	9.6.10^−10^	5.6.10^−54^	8.9.10^−08^
**RF4**	VP4–110	5NCR-719 – VP4–182	1.5.10^−15^	7.2.10^−16^	7.2.10^−19^	2.8.10^−06^	3.1.10^−06^	1.4.10^−25^	1.3.10^−02^
	2A-308	2A-248–2A-326	5.8.10^−20^	7.6.10^−18^	2.5.10^−20^	3.6.10^−08^	6.8.10^−05^	5.6.10^−54^	8.9.10^−08^
	3C-465	3C-361–3C-517	5.8.10^−20^	7.6.10^−18^	2.5.10^−20^	1.7.10^−18^	9.6.10^−10^	5.6.10^−54^	8.9.10^−08^
**RF-5**	VP4–6	5NCR-699 – VP4–51	9.2.10^−09^	2.2.10^−08^	8.0.10^−08^	9.2.10^−07^	1.2.10^−04^	1.6.10^−22^	9.9.10^−07^
	2A-308	2A-248–2A-326	5.8.10^−20^	7.6.10^−18^	2.5.10^−20^	3.6.10^−08^	6.8.10^−05^	5.6.10^−54^	8.9.10^−08^
	3C-506	3C-309–3C-524	2.8.10^−11^	1.8.10^−01^	1.1.10^−11^	1.3.10^−10^	1.5.10^−05^	6.1.10^−18^	5.5.10^−13^
**RF6**	VP4–64	5NCR-699 – VP4–71	3.3.10^−06^	2.1.10^−04^	1.2.10^−04^	2.9.10^−05^	3.0.10^−05^	2.3.10^−22^	/^**b**^
	2A-308	2A-248–2A-326	5.8.10^−20^	7.6.10^−18^	2.5.10^−20^	3.6.10^−08^	6.8.10^−05^	5.6.10^−54^	8.9.10^−08^
	3C-504	3C-309–3C-524	2.8.10^−11^	1.8.10^−01^	1.1.10^−11^	1.3.10^−10^	1.5.10^−05^	6.1.10^−18^	5.5.10^−13^
**RF7**	VP4–64	5NCR-699 – VP4–71	2.5.10^−06^	3.4.10^−07^	9.3.10^−05^	1.5.10^−05^	1.5.10^−05^	1.8.10^−22^	9.9.10^−07^
	2A-308	2A-248–2A-326	5.8.10^−20^	7.6.10^−18^	2.5.10^−20^	3.6.10^−08^	6.8.10^−05^	5.6.10^−54^	8.9.10^−08^
	2C-95	2C-66–2C-120	3.4.10^−20^	9.3.10^−61^	5.6.10^−66^	1.1.10^−34^	3.5.10^−14^	1.3.10^−50^	2.8.10^−68^

^a^RF3 differs from RF4 by the insertion of a fragment in gene 3C, located between positions 22 and 361 but not clearly determined by tools.

^b^Non-significant *P*-value.

Of the 57 AFP/AFM cases reported during the period studied, lineage assignment was feasible in 53 patients (93.0%) because genomic data were incomplete in the other patients (Supplementary Table S2). Most patients (*n =* 47) were infected by C1 viruses but precise lineage assignment was only possible in 16 patients. Within the C1 group, the C1.3/RF5 lineage was detected in 14 patients, 13 in Spain, and 1 in France. Two additional cases in the Czech Republic were linked to C1.3/RF9. No case was associated with C1.3/RF6, which has nevertheless circulated widely. A smaller number of AFP/AFM cases (France, *n =* 5; Spain, *n =* 1) were attributed to C2 viruses.

### 3.5. Evolutionary history, population dynamics, and geographic distribution of the enterovirus A71

**Figure 5:**
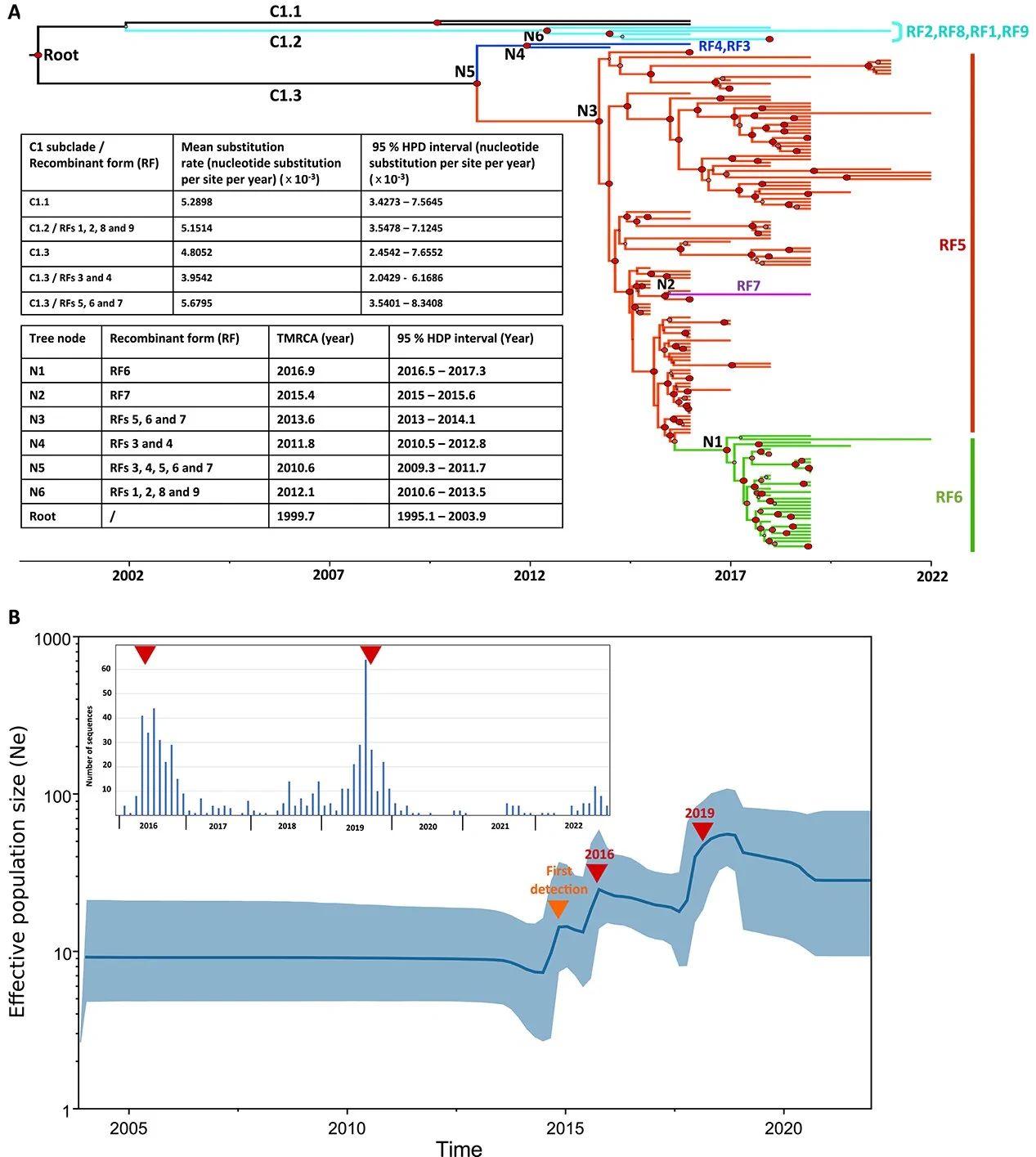
Evolutionary history and phylodynamic analyses of the EV-A71 C1 clade based on P1 genomic region. The evolutionary history of EV-A71 C1 was inferred using a MCC tree constructed from 161 P1 sequences with BEAST v2 under a relaxed molecular clock and the GTR substitution model, based on ten independent MCMC runs of one billion generations (a). Branches coloured by RFs and subclades. The number of sequences per RF is n=1 for RF1, RF2, RF3, RF4, RF7 and RF8; RF5 (n=92), RF6 (n=32), and RF9 (n=2). Node support is shown by circle size. Evolutionary rates and node ages, with 95% highest posterior density (HPD) intervals, are summarized in the inset tables. The effective population size of EV-A71 over time was inferred from the Bayesian skyline plot (b). The dark line denotes the median estimate, with the shaded area representing the 95% HPD interval. Arrows highlight periods of exponential growth. The number of clinically reported cases from 2016–2022 in this study is represented monthly in the inset panel, with arrowheads marking periods corresponding to increases in population size (2016 and 2019).

The phylogeny inferred for the P1 genomic region was supported by a strong molecular clock signal (Supplementary Fig. S4) and revealed the same three groupings corresponding to the C1.1, C1.2, and C1.3 subclades (Fig. 5a). The C1.2 subclade included RFs 1, 2, 8, and 9, whereas the C1.3 subclade included both sporadic RFs (3, 4, and 7) and epidemic RFs (5 and 6). Molecular clock analyses indicated that subclades C1.2 and C1.3 emerged between 2009 and 2014, while RF5 arose between 2013 and 2014, approximately one year prior to its first documented isolation in Germany; subsequently, RF6 and RF7 diverged from distinct lineages within the RF5 subclade. RF6 arose between 2016 and 2017, around two years before its first detection in France. Modelling of viral demography inferred with the P1 phylogeny (Fig. 5a) indicated a gradual increase in the effective population size of EV-A71 C1 from 2015 to 2022 (Fig. 5b). This expansion began with the emergence and circulation of C1.3/RF5 viruses. Two subsequent growth phases coincided with the spread of C1.3/RF5 (in 2016) and the expansion of C1.3/RF6 between 2016 and 2017. These phases of demographic growth are consistent with the outbreaks described above (Fig. 1b) and documented by the epidemiological surveillance of reported clinical cases in Germany in 2015 (Böttcher et al. 2016), Spain and France in 2016 (Casas-Alba et al. 2017, Ngangas et al. 2019), and France again in 2019 (this study).

**Figure 6:**
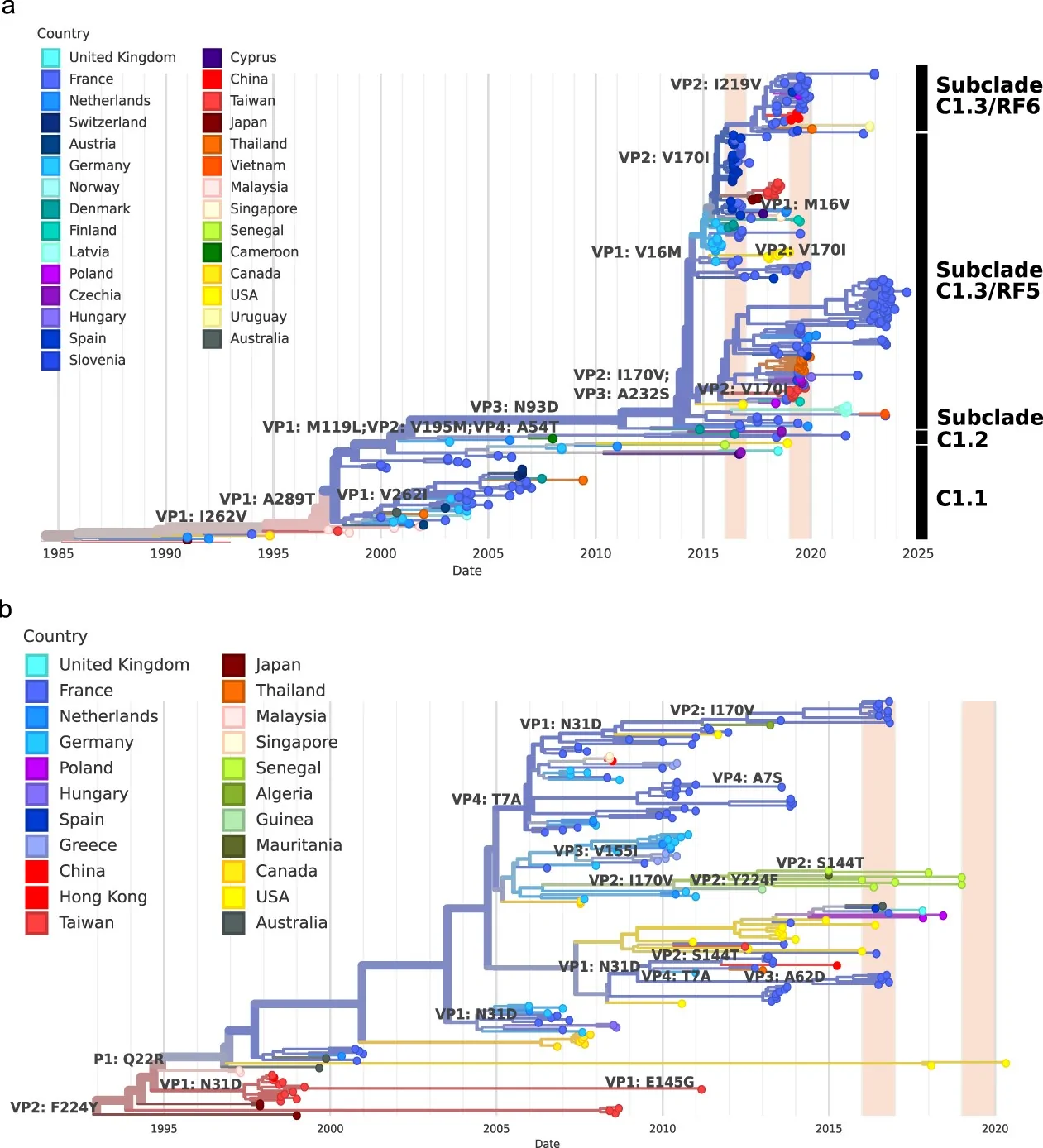
Time-resolved geographic distribution of EV-A71 C1 and C2 subclades visualized using Nextstrain. A maximum-likelihood phylogeny was reconstructed with Nextstrain from sequences of complete or near-complete genomes from this study (Table 1) and GenBank sequences for a total of 1975 P1 sequences sampled between 1975 and 2025. Two subtrees are presented: (a) one for subclade C1, showing 303 sequences sampled between 1991 and 2024; and (b) one for subclade C2, showing 175 sequences sampled between 1997 and 2020. Branches and tips are coloured by country of sampling, showing their internal structure and the fine-scale phylogeographic clustering. Backbone branches are coloured according to their inferred country of origin and labelled with percentage of confidence. The two epidemic periods (2016 and 2019) of EV-A71 circulation in Europe are highlighted with orange boxes. Amino acid changes were inferred and mapped onto the phylogenetic branches. The amino acid position was referenced on the BrCr reference genome (U22521.1).

To investigate global evolution and spread across countries, we placed the P1 sequences of our study among all publicly available EV-A71 P1 sequences (final dataset of 1975 sequences; nextstrain.org/groups/hodcroftlab/enterovirus/A71/P1) and reconstructed a time phylogeny using Nextstrain. Supplementary Fig. S5 shows the geographic distribution of the subset of C1 sequences and Fig. 6a a subtree of clade C1. The resulting tree clearly shows that subclades C1.2 and C1.3 originated from distinct C1.1 lineages (Fig. 6a). However, the precise origin of subclade C1.3 could not be inferred from the C1.1 sequences sampled between 2000 and 2010. Viral sequences were found in different countries between 2016 and 2022, and the extent of viral intermixing varied during this period (Table 5). In 2015–2016, the epidemic pattern was dominated by the 2016 outbreak in Spain associated with subclade C1.3/RF5, showing successive intermixing with viruses from Denmark, Germany, France, and the USA (Fig. 6a). With the 2019 upsurge in France, viral intermixing became more complex in 2018–2020, involving a set of co-circulating viruses belonging to the prevalent C1.3/RF5 and C1.3/RF6 subclades, detected in eight European countries and five non-European countries (Table 5; Supplementary Fig. S5). Sporadic detection of C1.3/RF7, C1.2/RF2, C1.2/RF9, and C1.1 lineages was also recorded during this period. Phylogenetic tree topology shows that most C1.3/RF5 lineages detected in 2019 derived from previously unreported 2016 lineages that persisted during the inter-epidemic period. Although viruses from subclades C1.1 and C1.2 were rarely detected during the study period and contributed little to epidemic transmission, their diverse geographic origins confirm extensive cross-country migration (Table 5).

**Table 5:** Geographic distribution of EV-A71 C1 variants between 2015 and 2022 based on Nextstrain data.

Period	EV-A71 subclade	European country	Non-European country
01/2015–12/2016^a^	C1.3/RF5	Denmark, France, Germany, Spain	USA
	C1.2/RF1	Denmark	/
	C1.1	Cyprus, Czech Republic	Senegal
2017	C1.3/RF5	Cyprus, France, Portugal	Japan
01/2018–04/2020^b^	C1.3/RF5	Czech Republic, Finland, France, Hungary, Netherlands, Poland, Slovenia, Spain,	Thailand, USA, Singapore, Taiwan
	C1.3/RF6	France, Poland, Portugal, Slovenia, Spain	China, Thailand
	C1.3/RF7	France	/
	C1.2/RF2, RF9	Czech Republic, France	/
	C1.1	United Kingdom	USA
04/2020–12/2022	C1.3/RF5	France, Latvia, Portugal	/
	C1.3/RF6	France	Uruguay
	C1.2/RF8	France	/

^a^Outbreak of encephalitis in Spain in 2016.

^b^Upsurge of neurological diseases in France in 2019.

The few subgenogroup C2 viruses detected between 2016 and 2022 primarily contributed to the 2016 epidemic and subsequently persisted during the inter-epidemic period (Fig. 6b). Phylogenetic analysis showed four distinct lineages distributed across European countries, with clear evidence of geographic mixing. No signs of recombination were detected, and no large-scale epidemic was reported in Europe during the study period.

### 3.6. Amino acid substitutions in capsid proteins among enterovirus A71 subclades

Thirteen amino acid substitutions, including three reversions, were identified during the evolution of subgenogroup C1 sequences using Nextstrain phylogenetic ancestral reconstruction (Fig. 6a and Supplementary Table S3). Along the main tree backbone leading to subclade C1.3, seven amino acid substitutions occurred sequentially: VP1-I262V, VP1-A289T, VP3-N93D, VP3-A232S, VP2-I170V, VP1-V16M, and VP2-I219V. VP1-I262V and VP1-A289T occurred near the base of the phylogenetic tree and are shared by all C1 sequences. Site VP1-262 is positioned on the βI strand and located within the EV-A71 capsid core (Supplementary Fig. S6). The I262V substitution is a conservative change expected to have minimal structural consequences. A reversion at VP1-262 was restricted to a C1.1 lineage. Because alanine is weakly hydrophobic whereas threonine is polar, the A289T substitution may induce local conformational adjustments in the VP1 C-terminus, which is exposed on the capsid surface (Supplementary Fig. S6).

**Figure 7:**
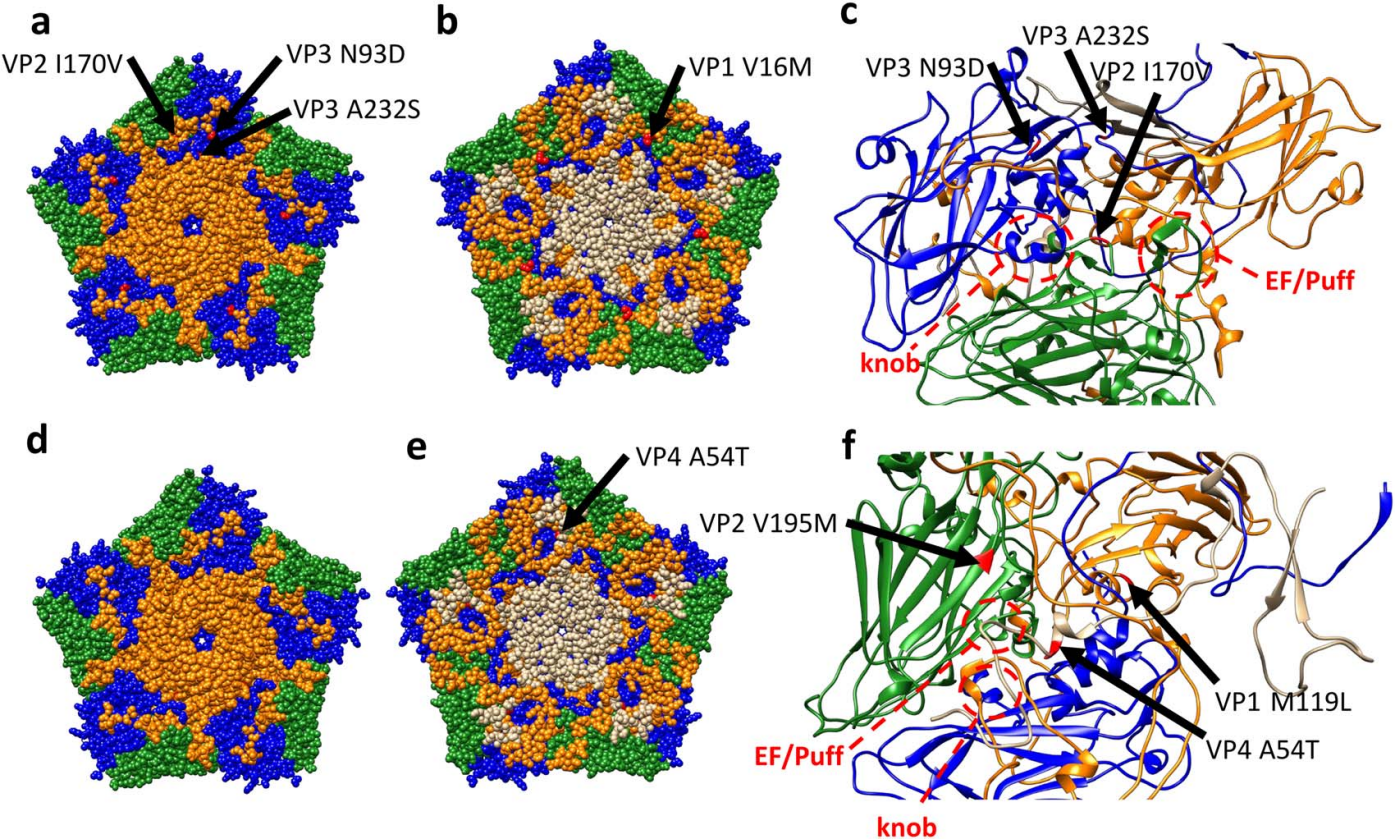
Structural modelling of the EV-A71 capsid pentamer for subclades C1.2 and C1.3. Representative reference sequences for subclades C1.2 (MG367600) and C1.3 (LR027531), selected based on the absence of minor amino acid variations relative to other sequences within each subclade, were used to generate capsid pentamer models using AlphaFold. The predicted models were validated by comparison with the available crystal structure (PDB ID:8E2X). Structural representations were generated using UCSF Chimera. Capsid proteins are shown in different colours: VP1 (orange), VP2 (green), VP3 (blue), and VP4 (grey). Amino acid substitutions inferred by Nextstrain are highlighted in red. Surface-exposed mutations are indicated by arrows (panels a, b, d, and e), whereas buried substitutions are not shown (VP1:M119L and VP2:V195M are therefore not included in these panels). Panels a–c correspond to subclade C1.3, and panels d–f to subclade C1.2. Panels a and d show external views of the capsid pentamer, while panels b and e display internal views. Panels c and f present enlarged views highlighting the spatial proximity of mutations within the capsid structure and their localization within β-sheets and α-helices.

The four amino acid substitutions VP3-N93D, VP3-A232S, VP2-I170V, and VP1-V16M characterize the evolution of subclade C1.3/RF5 (Fig. 7a–c). In 3D maps, VP3-93 lies in a turn below the VP3 knob loop, while VP3-232 is located within the C-terminus, directed towards the neighbouring VP1 protein on the inner side of the outer shell. Both residues occupy the same surface-proximal region of VP3. The VP3-N93D change introduces a negative charge, while VP3-A232S adds a polar hydroxyl group to the C1.3 VP3 protein. The VP2-I170V substitution is located beneath the outer surface of the capsid and induces no change in charge. A reversion at VP2-170 occurred in eight distinct C1.3/RF5 clusters containing a limited number of sequences. VP1-16 lies on the interior surface of the capsid and is in close contact with the viral genome. VP2-I219V is a conservative non-polar change within the βH strand expected to be solvent-exposed, which occurred only in a subset of C1.3/RF6 sequences.

Three amino acid substitutions characterize the evolutionary pattern of subclade C1.2: VP4-A54T, VP2-V195M, and VP1-M119L (Fig. 6a). Structural modelling indicated that the three residues are positioned within the thickness of the capsid wall, rather than exposed on the inner or outer surfaces (Fig. 7d–f). The A54T substitution introduces a polar residue into the C1.2 VP4 chain. The V195M change is conservative in terms of charge, but increases the side-chain bulk and flexibility of the C1.2 VP2 chain. VP1–119 is located within an alpha helix near the hydrophobic pocket beneath the VP1 loops. The M119L substitution is associated with a modest reduction in side-chain bulk within the C1.2 capsid.

The Nextstrain phylogeny showed 10 amino acid substitutions among the C2 sequences (Fig. 6b). Two changes occurred near the base of the tree (VP2-F224Y, VP1-Q22R), whereas VP4-T7A was detected only in a subset of C2 viruses circulating after 2005. Two substitutions occurred independently on separate lineages: VP1-N31D appeared three times, and VP2-I170V occurred twice.

## 4. Discussion

This large multi-country study provides a comprehensive overview of the burden and clinical spectrum of EV-A71 infections recorded retrospectively from 19 institutes in 14 European countries between 2016 and 2022. During this period, two epidemic upsurges occurred in 2016 and 2019 (Böttcher et al. 2016, Casas-Alba et al. 2017, Ngangas et al. 2019). The increase in EV-A71 cases in European countries was dominated by C1 subgenogroup viruses, with C2 viruses being less frequently detected and C4 viruses only sporadically. Most infection cases (86.2% of patients) were caused by subclade C1.3 viruses. The clinical spectrum dominated by neurological involvement confirms that EV-A71 is a major cause of paediatric infectious disease in Europe (Harvala et al. 2025). One of the main findings from the clinical data in our patient cohort is the high incidence of infection among children under two years of age and the frequent occurrence of a neurological disease, with meningitis being reported in 14.9% of patients, encephalitis in 23.1%. Our survey also documents a substantial number of AFP/AFM cases across five countries, totalling 57 patients (10.6%.). Most infections were caused by C1 viruses with fewer cases involving C2 viruses. The absence of AFP/AFM cases associated with C1.3/RF6, despite its extensive circulation, may suggest a different pattern of neurological involvement. However, the limited number of complete genomes available does not allow any definitive conclusion regarding differences in neurological pathogenicity between RFs. The spectrum of neurological manifestations observed is consistent with that reported during the 2018 outbreak of EV-A71 subgenogroup C1 in children in Colorado, USA (Messacar et al. 2020). Notably, the proportion of hospitalized patients requiring intensive care (15.7%) highlights the persistent risk of severe outcomes associated with EV-A71 C1 infections (Huang et al. 2020, Kapadia et al. 2021, Akinnurun et al. 2023, Nieminen et al. 2024, David et al. 2025) and underlines its ongoing relevance to public health. Although EV-A71 remains a leading cause of HFMD (Martínez-López et al. 2021, Puenpa et al. 2021, Zhou et al. 2021), only 31.3% of patients in our cohort had HFMD or mucocutaneous manifestations, and half of these cases were ambulatory patients (*n =* 73/168). Our findings and those from other studies emphasize the importance of maintaining EV-A71 surveillance within paediatric infectious disease surveillance systems in Europe (Wörner et al. 2021, Harvala et al. 2025). The virus should also be monitored in primary care, given that HFMD cases are not given priority for hospital attendance.

Phylodynamic analyses of viral genomic sequences showed a sustained increase in the effective population size of C1 viruses from 2015 onwards, with distinct phases of accelerated growth coinciding with the 2015 emergence (in Germany), the 2016 outbreak (Spain, France), and the 2019 outbreak (France). This timing of EV-A71 circulation and the emergence of subclade C1.3, which was assessed between 2010 and 2013 shortly before its first detection, suggest that this virus population had already spread across Europe by the time the first spikes in clinical cases were reported. These findings reinforce the need for early genomic detection of newly emerging EV-A71 lineages, so as to provide advanced warning of ensuing waves in clinical incidence, as has been reported for other epidemic viruses (Link-Gelles et al. 2022, Grapin et al. 2023). The genomic sequence data also indicate the importance of recombination in EV-A71 C1 virus diversification, with nine RFs being detected, including four that had not been reported previously. The number of EV-A71 cases and the use of high-throughput sequencing explain why six RFs were detected in France, whereas sequencing data from other European countries have so far identified RF1 and RF3 in Denmark (Midgley et al. 2017) and RF9 in the Czech Republic (this study). Whole-genome similarity analyses revealed mosaic recombination patterns with breakpoints primarily located at the 5′NCR terminus and within the P2 and P3 genomic regions. These findings are consistent with previously reported data (Nikolaidis et al. 2019). However, accurate determination of the evolutionary origin of recombinant EV-A71 lineages is complicated by a twofold sampling problem. First, the precise origin of subclade C1.3 viruses cannot be resolved from C1.1 sequences collected between 2000 and 2010, likely due to incomplete historical sampling. The paucity of genome sequences from this period may have resulted in missing evolutionary intermediates, thereby reducing phylogenetic resolution of early divergence events. Second, identifying the parental lineages of C1.3 RFs involves the added difficulty of requiring genomic data from other types of the *Enterovirus alphacoxsackie* species, which co-circulated with the EV-A71 C1.1 strains across broad geographic and temporal scales, which is beyond the scope of the study. Further investigations are required to overcome these constraints. The number of C1 RFs detected within the patient cohort in less than a decade is remarkable, suggesting that, unlike the C4 virus in Asia, C1 is undergoing an unprecedented level of recombination. Subgenogroup C4, which is endemic in the Asia-Pacific region and responsible for large-scale HFMD outbreaks affecting millions of people, has generated comparatively fewer recombinant lineages (Lee et al. 2018). This discrepancy is unlikely to result from sampling bias given the extensive surveillance or mandatory HFMD reporting implemented in several Asian countries (Chiu et al. 2020, Liu et al. 2025).

Of the various recombinant lineages identified in this study, those from subclades C1.3/RF5 and C1.3/RF6 were the most prevalent, spreading across nine and four European countries, respectively. Both RFs also spread beyond Europe, and C1.3/RF5 was found in five additional countries (the USA, Japan, Singapore, Thailand, and Taiwan), while the more recent C1.3/RF6 was found in three additional countries (continental China, Thailand, and Uruguay). Overall, these findings suggest that Europe has become a source and sink of epidemic C1.3 recombinant lineages, and the persistence and the epidemic circulation of C1.3/RF5 and C1.3/RF6 evidence their epidemiological fitness compared to that of the other RFs. Recombination is a major driver of viral evolution and epidemiology, acting alongside high mutation rates and selection as a key adaptive process in many positive-strand RNA viruses (Bentley and Evans 2018, Bentley et al. 2021). Although we lack direct experimental evidence for a selective advantage among EV-A71 C1 RFs, the extensive genomic rearrangements in RF5 and RF6—particularly in the 5′UTR and 3D polymerase regions—suggest that these variants may have acquired beneficial mutations or purged deleterious ones (Xiao et al. 2016). For example, during the first well-documented outbreak of vaccine-derived paralysis, four independent type 1 vaccine-derived recombinant polioviruses were identified (Kew et al. 2002); these carried the 5′UTR and capsid regions of Sabin type 1 recombined with non-structural region fragments from other enterovirus C species members (now known as *Enterovirus coxsackiepol*). Our findings further demonstrate that recombination was a hallmark of the emergence of several lineages within EV-A71 subgenogroup C1, including C1.3, consistent with previous observations that EV-A71 genogroups and many subgenogroups originated from recombination events immediately preceding lineage expansion and outbreaks (McWilliam Leitch et al. 2012). The epidemiological success of viral lineages is therefore shaped not only by viral genetic processes and virus population structures within infected cells and hosts, but also by broad factors such as virus circulation through population movements, founder effects, and host population immunity (Dábilla and Dolan 2024, Khurana et al. 2026). Thus, the global expansion of RF5 and RF6 reflects a complex interplay between viral evolution and epidemiological context.

The capsid of C1 viruses accumulated relatively few amino acid substitutions compared with the extensive genomic changes driven by recombination. However, these changes could still have contributed to the epidemiological success of subclade C1.3. Within this subclade, two VP3 substitutions (N93D and A232S) are located on the external capsid surface but outside the major ‘knob’ loop containing dominant epitopes (Plevka et al. 2012). Although structural modelling alone does not allow direct inference of antigenic or phenotypic consequences, these surface-exposed residues could modulate interactions with neutralizing antibodies, which could in turn influence transmission dynamics. The VP1–V16M substitution, also characteristic of C1.3, lies within the N-terminus (residues 1–71), a domain implicated in viral uncoating and genome release (Lyu et al. 2014, Zhang et al. 2026), raising the possibility that it may affect early steps of viral replication. With the exception of the A289T change found in the VP1 C-terminus, the other amino acid substitutions detected in the multirecombinant C1 viruses that have been circulating since 2015 did not arise within the known EV-A71 antigenic epitopes (Plevka et al. 2012, Huang et al. 2015). This is in stark contrast to the evolutionary pattern of EV-D68, where continuous antigenic evolution is observed in the surface proteins, particularly in the hypervariable BC-loop (amino acids 90–103) and DE-loop (amino acids 140–148) of the VP1 protein, which contain receptor-binding sites and neutralizing epitopes (Hodcroft et al. 2022, Andrés et al. 2026). However, critically, C1 virus strains belonging to subclade C1.3, were reported to be circulating in Guangdong, China in 2018 and 2019—two years after vaccination campaigns began using C4-based vaccines (Zeng et al. 2021). These data suggest that C4-based vaccines do not provide complete cross-protection against C1 virus infections, especially since C1 viruses showed reduced neutralization by human sera from patients infected with B5 or C4 viruses, which in turn suggests that the C1 subgenogroup can partially evade pre-existing immunity in the population (Yang et al. 2022, Liu et al. 2025). Our study was not designed to directly compare antigenic differences but sequence comparisons demonstrate that C4 and historical C1.1 viruses share identical residues at VP3 positions 93 and 232. This observation supports the hypothesis that antibodies elicited by C4 viruses may exhibit reduced recognition of C1.3 viruses harbouring amino acid changes at these positions. However, the precise impact on C4 vaccine-induced cross-protection against C1.1 and C1.3 viruses requires further investigation through dedicated neutralization assays. Although an EV-A71 vaccination coverage above 20% was shown to be effective in preventing severe EV-A71 HFMD (Liu et al. 2025), lower coverage levels, as observed in Southwest China (Yang et al. 2022), might be insufficient to exert population-level immunity to prevent the spread of divergent C1 viruses. Between 2018 and early 2019, Taiwan experienced an outbreak of HFMD with neurological complications, primarily caused by EV-A71 C1 (Huang et al. 2020). The emergence of C1 viruses (related to subclade C1.3) in the unvaccinated Taiwanese paediatric population, marked a notable shift in EV-A71 circulating strains, which were historically dominated by B5 and C4 viruses. EV-A71 subgenogroup C1 (subclade C1.3) has also emerged as a cause of HFMD in Thailand alongside the subgenogroups B5, C2, C4, and C5 (Puenpa et al. 2021). The emergence of C1 has been associated with an increased incidence of severe neurological complications in children. In Taiwan and Thailand, there are currently no consolidated data on vaccination coverage (Liu et al. 2025), which makes it difficult to accurately assess the impact of vaccination strategies in these settings. In Uruguay, the first detection of EV-A71 C1 was also associated with severe neurological complications in two children (Lizasoain et al. 2024). Genetic analysis confirmed that these strains are closely related to European C1.3 viruses and not to local South American strains.

As has been reported for other EV types, such as EV-D68 and CV-A6 (Hodcroft et al. 2022, Tomba Ngangas et al. 2022), the presence of multiple co-circulating EV-A71 C1 lineages in different countries illustrates that this virus has expanded in geographically interconnected settings, where importations and local amplification are closely interwoven. These findings underscore the critical role of genetic and antigenic surveillance in guiding vaccine development and public health responses. The emergence of subclade C1.3 highlights the need for continuous assessment of antigenic differences among circulating EV-A71 lineages. Although current evidence suggests that C4-based vaccines may confer cross-protection against diverse EV-A71 strains—including non-C4 lineages—the characteristic VP3 substitutions of C1.3 and its post-vaccination emergence in Taiwan and China raise concerns about real-world efficacy. The potential antigenic mismatch between C4-based vaccines and C1.3 thus warrants immunogenicity studies using contemporary isolates to determine the breadth of protective efficacy. A phased approach, beginning with targeted pilot programmes in European countries, would enable balanced evaluation of public health benefits against remaining uncertainties.

This study has limitations. The data were obtained retrospectively from European countries that do not have harmonized surveillance systems and diagnostic testing for EV infections (Harvala et al. 2018, de Schrijver et al. 2025, Hirvonen et al. 2025) and consequently, the number of EV-A71 cases may be underestimated by potential differences in diagnostic sensitivity, surveillance intensity, and sequencing capacity among participating institutions. Furthermore, the clinical presentation of EV-A71 cases may be biassed by the fact that infected children and severe cases are more likely to be investigated and hospitalized, respectively. This could result in neurological complications being overrepresented in children and HFMD cases being underestimated, since HFMD monitoring is currently only implemented only in France. The sequence dataset is also constrained by heterogeneous diagnostic practices, variable sampling intensity between countries and over time, and the availability of residual clinical material for sequencing. These factors can bias estimates of lineage prevalence, geographic origins, and demographic histories. The lack of systematic whole-genome sequencing across Europe has likely resulted in an underestimation of the diversity and circulation of EV-A71 lineages. This underestimation could have biassed phylogeographic analyses, which are primarily descriptive and cannot attribute differences in clinical severity to specific genetic changes or RFs directly. Despite these limitations, the study outlines the recent evolutionary trajectories of EV-A71 C1 viruses and shows how recombination, lineage diversification, and regional seeding events have shaped the current epidemiological landscape of EV-A71 infections in Europe.

In conclusion, the study shows that there was a high proportion of AFP/AFM cases among EV-A71 patients in European countries following the emergence and spread of the multirecombinant lineages C1.3/RF5 and C1.3/RF6 between 2016 and 2022. From a public health perspective, the findings highlight three priorities: (i) sustained molecular surveillance of EV-A71, ideally integrating VP1 and whole-genome sequencing, is essential to detect the emergence of new RFs and to monitor shifts in lineage dominance over time; (ii) the association of C1.3/RF5 and C1.3/RF6 with neurological disease in young children highlights the need for standardized clinical data collection and harmonized case definitions across European surveillance networks; and (iii) the demonstration that Europe can serve both as a sink and a source of epidemic C1 lineages emphasizes the importance of tighter international data sharing and coordinated risk assessments, particularly in the context of potential vaccine introduction.

## Members of the French Enterovirus Surveillance Network

Laurent Andreoletti, CHU Reims, Laboratoire de Virologie, Reims; Christelle Auvray, CHU Dijon Bourgogne, Laboratoire de Virologie-Sérologie, Dijon; Thomas Bourlet, CHU de Saint-Etienne, Service des agents infectieux et d’hygiène, Saint-Etienne; Eline Bremond, CHU de Brest, Unité de Virologie, Brest; Céline Bressollette Bodin, CHU Nantes, Service de Virologie, Hôtel Dieu, Nantes; Véronique Brodard, CHU Reims, Laboratoire de Virologie, Reims; Marianne Burgard, Hôpital Necker-Enfants Malades, AP-HP, Laboratoire de Virologie, Paris; Sonia Burrel, GH Pellegrin, Université de Bordeaux, Service de Virologie, Bordeaux; CNRS UMR 5234, Fundamental Microbiology and Pathogenicity, Bordeaux. Anne Cady, CH Brocéliande Atlantique, Laboratoire de Biologie Médicale, Vannes. Alexis de Rougemont, CHU Dijon Bourgogne, Laboratoire de Virologie-Sérologie, Dijon. Cyril Debuysschere, CHU de Lille, Laboratoire de Virologie ULR, Univ Lille, Lille. Laura Djamdjian, CH de Gonesse, Laboratoire de Biologie Médicale, Gonesse; Violaine Doat, CH Bourgoin-Jallieu, Laboratoire de Biologie Médicale, Bourgoin-Jallieu; Joséphine Dorin, CH Antibes-Juan-les-Pins, Laboratoire de Biologie médicale, Antibes; Thomas Drumel, CHU Nantes, Service de Virologie, Hôtel Dieu, Nantes; Alexandra Ducancelle, CHU d’Angers, Laboratoire de virologie, Département de Biologie des Agents Infectieux, Angers; Ilka Engelmann, CHU Montpellier, Site Unique de Biologie, Laboratoire de Virologie, Montpellier; Eric Farfour, Hôpital Foch, Service de biologie clinique, Suresnes; Vincent Foulongne, CHU Montpellier, Site Unique de Biologie, Laboratoire de Virologie, Montpellier; Agathe Francart, CH Libourne Robert Moulin, Laboratoire de Biologie Médicale, Libourne; Floriane Gallais, CHU Strasbourg, Laboratoire de virologie, Strasbourg; Magali Garcia, CHU de Poitiers, Laboratoire de virologie et mycobactériologie, Poitiers; Catherine Gaudy-Graffin, CHRU de Tours, Unité Emergence, Service de bactériologie, virologie et hygiène hospitalière, Tours; Mathilde Gay, GH Pellegrin, Université de Bordeaux, Service de Virologie, Bordeaux, CNRS UMR 5234, Fundamental Microbiology and Pathogenicity, Bordeaux; Juliette Gillon, CHI Frejus, Frejus; Geraldine Gonfrier, CHU de Nice, Laboratoire de Virologie Archet 2, Nice; Marie Gueudin, CHU Rouen, Laboratoire de Virologie, Rouen; Pascal, Guiet, CH Montargis, Laboratoire de Biologie Médicale, Amilly; Clémence, Guillaume, CHU d’Orléans, Laboratoire de Microbiologie, Orléans; Lynda Handala, CHRU de Tours, Unité Emergence, Service de bactériologie, virologie et hygiène hospitalière, Tours; Anne Christine Jaouen, CH de la Côte Basque, Laboratoire de Biologie médicale, Bayonne; Marine Jourdain, Hôpitaux Nord-Ouest, Villefranche sur Saône; Gisèle Lagathu, CHU Rennes, Laboratoire de Virologie, Rennes; Sylvie Larrat, CHU Grenoble-Alpes, Laboratoire de Virologie, Institut de Biologie et Pathologie, Grenoble; Mouna Lazrek, CHU de Lille, Laboratoire de Virologie ULR, Univ Lille, Lille; Caroline LEFEUVRE, CHU d’Angers, Laboratoire de virologie, Département de Biologie des Agents Infectieux, Angers; Véronique Lemee, CHU Rouen, Laboratoire de Virologie, Rouen; Quentin LepilleR, CHU de Besançon, Laboratoire de Virologie, Besançon; Nicolas Leveque, CHU de Poitiers, Laboratoire de virologie et mycobactériologie, Poitiers; David Leyssene, CH de la Côte Basque, Laboratoire de Biologie médicale, Bayonne; Anne-Sophie L’Honneur, CHU Cochin, APHP, Service de virologie, Paris; Marie Louchet-Ducoroy, CHU Amiens, Laboratoire de Virologie, Amiens; Léa Luciani, CHU Marseille, Laboratoire de virologie aigue et tropicale, LAI AP-HM, Marseille; Sarah Mafi, CHU Limoges, Laboratoire de Virologie, Limoges; Alice-Andrée Mariaggi, CHU Cochin, APHP, Service de virologie, Paris; Victoria Marie, CHU d’Orléans, Laboratoire de Microbiologie, Orléans; Stéphanie Marque Juillet, CH de Versailles, Laboratoire de Biologie Médicale, Le Chesnay; Solène Marty-Quinternet, CHU de Besançon, Laboratoire de Virologie, Besançon; Soraya Metref, CH de Gonesse, Laboratoire de Biologie Médicale, Gonesse; Yanne Michel, CHU St Antoine–Tenon–Trousseau, AP-HP, Laboratoire de Virologie, Paris; Marion Migueres, CHU de Toulouse, Laboratoire de Virologie, Toulouse; Menel Mohamedi, Hôpital Saint Louis, APHP, Laboratoire de Virologie, Paris; Jean-Benjamin Murat, CH de Roanne, Laboratoire de Biologie médicale, Roanne; Sylvie Pillet, CHU de Saint-Etienne, Service des agents infectieux et d’hygiène, Saint-Etienne; Léa Pilorge, CHU de Brest, Unité de Virologie, Brest; Emeline Riverain, CH François Quesnay, Laboratoire de Biologie médicale, Mantes la Jolie; Sylvie Rogez, CHU Limoges, Laboratoire de Virologie, Limoges; Maud Salmona, Hôpital Saint Louis, APHP, Laboratoire de Virologie, Paris; Cécile Schanen, CHU de Caen, Service de Virologie, Caen; Aurélie Schnuriger, CHU St Antoine–Tenon–Trousseau, AP-HP, Laboratoire de Virologie, Paris; Evelyne, Schvoerer, CHRU de Nancy, Laboratoire de virologie, Dpt microbiologie, Vandeouvre-les-Nancy; Morgane Solis, CHU Strasbourg, Laboratoire de virologie, Strasbourg; Alexandra Teboul, CH de Versailles, Laboratoire de Biologie Médicale, Le Chesnay; Charlotte Tellini, CH Bourgoin-Jallieu, Laboratoire de Biologie Médicale, Bourgoin-Jallieu; Vincent Thibault, CHU Rennes, Laboratoire de Virologie, Rennes; Anne-Lise Toyer CHI Toulon, Laboratoire de Biologie Médicale, Toulon; Nicolas Traversier, CHU Réunion, Laboratoire de microbiologie, St Denis; Pauline Tremeaux, CHU de Toulouse, Laboratoire de Virologie, Toulouse; Aurélie truffot, CHU Grenoble-Alpes, Laboratoire de Virologie, Institut de Biologie et Pathologie, Grenoble; Caroline Vaudron, CH Libourne Robert Moulin, Laboratoire de Biologie Médicale, Libourne; Véronique Venard, CHRU de Nancy, Laboratoire de virologie, Dpt microbiologie, Vandeouvre-les-Nancy; Nicolas Veyrenche, Hôpital Necker-Enfants Malades, AP-HP, Laboratoire de Virologie, Paris.

## Supplementary Material

Supplementary_materials_veag051
