# Supplementary material for: Emergence and spread of multirecombinant enterovirus A71 lineages C1.3/RF5 and C1.3/RF6 associated with neurological diseases in Europe, 2016–2022, ENPEN multicentre study
Source: Virus Evol. 2026 Aug 8;12(1):veag051. doi: 10.1093/ve/veag051 (PMC13580226; doi:10.1093/ve/veag051)
Supplement: Supplementary file 1 [file supplementary_materials_veag051.pdf]

SUPPLEMENTARY DATA

Emergence and spread of multirecombinant enterovirus A71 lineages C1.3/RF5 and C1.3/RF6 associated with neurological diseases in Europe, 2016–2022

Belele Siméon Bakyono[1], Audrey Mirand[1,2], Nadia Neuner-Jehle[3], Cristina Andrés[4], Jorgina Vila[5,6], Andrés Antón[4,7], Isabelle Schuffenecker[8], Marion Jeannoël[8], Laurence Josset[8,9], Elisabeth Toverud Landaas[10], Suzanne Dudman[10], Mária Takács[11,12], Erika Bujáki[13], Ágnes Farkas[11], Laura Pellegrinelli[14], Sandro Binda[14], Sara Uceda Renteria[15], Jacky Flipse[16,17], Caroline M.A. Swanink[16], Jaco J. Verweij[18], Jean-Luc Murk[18], Karen Couderé[18], Magdalena Wieczorek[19], Kacper Toczylowski[20], Artur Sulik[20], Maria Milagrosa Montes Ros[21], Luis Piñeiro[21], Eider Oñate Vergara[22], Iñigo Ansa Isasa[21], Jan Richter[23], Christina Christodoulou[23], Ondřej Cinek[24], Kateřina Chudá[24], Petr Hubáček[24], Antonio Piralla[25], Federica A.M. Giardina[26], Paula Palminha[27], Raquel Neves[27], Carlos Ribeiro[27], Maja M. Lunar[28], Mario Poljak[28], Anne J. Jääskeläinen[29], Baiba Niedre-Otomere[30], the French Enterovirus Surveillance Network, Cécile Henquell[1,2], Emma B. Hodcroft[3], Sofie E Midgley[31], Natasa Berginc[32], Thea K. Fischer[33], Heli Harvala[34], Kimberley Benschop[35], Jean-Luc Bailly[1,*], Maxime Bisseux[1,2,*]

Affiliations

[1]Laboratoire Microorganismes, Génome et Environnement (LMGE), Université Clermont Auvergne, UMR CNRS 6023 - France

[2]Laboratoire de Virologie, CNR des Entérovirus et Parechovirus, CHU de Clermont-Ferrand, France

[3]Swiss Tropical and Public Health Institute, Allschwil, Switzerland, University of Basel, Basel, Switzerland; Swiss Institute of Bioinformatics, Lausanne, Switzerland

[4]Respiratory Viruses Unit, Microbiology Department, Vall d'Hebron Hospital Universitari, Vall d'Hebron Institut de Recerca (VHIR), Vall d'Hebron Barcelona Hospital Campus, Spain

[5]Pediatric Hospitalization Unit, Hospital Infantil i de la Dona Vall d'Hebron, Vall d'Hebron Barcelona Hospital Campus, Barcelona, Catalonia, Spain

[6]Infection and Immunity in Pediatric Patients Research Group, Vall d'Hebron Institut de Recerca (VHIR), Barcelona, Catalonia, Spain

[7]Biomedical Research Networking Center in Infectious Diseases, Instituto de Salud Carlos III (ISCIII), Madrid, Spain

[8]Centre National de Référence des Enterovirus et Parechovirus - Laboratoire associé, Institut des Agents Infectieux, Hospices Civils de Lyon, Lyon, France

[9]Plateforme de Séquençage des Pathogènes (genEPII), Hospices Civils de Lyon, Lyon, France

[10]Department of Microbiology, Oslo University Hospital; Institute of Clinical Medicine, University of Oslo, Oslo, Norway

[11]National Centre for Public Health and Pharmacy, Department of Virology, 1097 Budapest, Hungary

[12]Semmelweis University, Institute of Medical Microbiology, 1089, Budapest, Hungary

[13]Medicines and Healthcare products Regulatory Agency, Blanche Lane, South Mimms, EN6 3QG, United Kingdom

[14]Department of biomedical sciences for health, University of Milan, Milan, Italy

[15]Microbiology and Virology Unit, Foundation IRCCS Ca' Granda Ospedale Maggiore Policlinico, Milan, Italy

[16]Department of Medical Microbiology and Immunology, Rijnstate hospital, Arnhem, The Netherlands

[17]Laboratory for Medical Microbiology and Immunology, Dicoon, Elst, The Netherlands

[18]Microvida Laboratory of Medical Microbiology and Immunology, Elisabeth-Tweesteden Hospital, Tilburg, The Netherlands

[19] Department of Virology, National Institute of Public Health NIH - National Research Institute, Warsaw, Poland

[20] Department of Pediatric Infectious Diseases, Medical University of Bialystok, Poland

[21]Microbiology Department, Donostia University Hospital, Biogipuzkoa Health Research Institute, San Sebastián, Gipuzkoa, Spain

[22]Paedriatric Department, Donostia University Hospital, Biogipuzkoa Health Research Institute, San Sebastián, Gipuzkoa, Spain

[23]Department of Molecular Virology, Cyprus Institute of Neurology and Genetics, Nicosia, Cyprus

[24]Second Faculty of Medicine, Charles University in Prague, University Hospital Motol and Homolka, Prague, Czech Republic

[25]Microbiology and Virology Department, Fondazione IRCCS Policlinico San Matteo, Pavia, Italy

[26]Department of Clinical, Surgical, Diagnostic and Pediatric Sciences, University of Pavia, Pavia, Italy

[27]National Reference Laboratory for Vaccine Preventable Diseases, Department of Infectious Diseases, National Institute of Health Doutor Ricardo Jorge (INSA), Lisbon, Portugal

[28]Institute of Microbiology and Immunology, Faculty of Medicine, University of Ljubljana, Ljubljana, Slovenia

[29]University of Helsinki and Helsinki University Hospital, HUS Diagnostic Center, Virology and Immunology, Helsinki, Finland

[30]National Microbiology Reference Laboratory of Latvia, Laboratory “Latvian Centre of Infectious Diseases”, Laboratory Service, Riga East University Hospital, Riga, Latvia

[31]Dept of Virology & Mircrobiology Preparedness, Statens Serum Institut, Copenhagen, Denmark

[32]National Laboratory of Health, Environment and Food, Laboratory for Public Health Virology, Ljubljana, Slovenia

[33]Dept Clinical Research, Nordsjaellands Hospital, Hilleroed, Denmark; University of Copenhagen, Copenhagen, Denmark

[34]Institute of Biomedicine, Faculty of Medicine; Medical Microbiology, Turku University Hospital, Turku, Finland

[35]Centre for Infectious Disease Control (CIb), Centre for Infectious Disease Research, Diagnostics and laboratory Surveillance (IDS) Dutch National Institute for Public Health and the Environment, Bilthoven, The Netherlands

TABLE OF CONTENTS

Supplementary methods 4

Sample processing and nucleic acid extraction 4

PCR amplification of complete genomes 4

Sequencing protocol of the Clermont-Ferrand laboratory (France) 4

Supplementary results... 6

Figure S1. Complete workflow of data analyses performed in the study. 6

Figure S2. Phylogenetic clustering of EV-A71 VP1 sequences and assignment to extant genogroups. 8

Figure S3. Nucleotide similarity patterns of complete EV-A71 genomes determined with Simplot and assignment to recombinant forms (RF). 12

Figure S4. Assessment of the molecular clock of the P1 genomic region of EV-A71. 13

Figure S5. Visualisation of the geographic distribution of EV-A71 C1 subclades using Nextstrain......................14

Figure S6. Localisation of the two common mutations on all C1 lineages (VP1: 262 and 289) on the structural modeling of the EV-A71 capsid pentamer...15

Table S1. GenBank accession numbers of complete genomes used in this study. 16

Table S2. Relationship between AFP/AFM cases and EV-A71 subgenogroups and Recombinant forms. 19

Table S3. Comparison of amino acid substitutions in EV-A71 subclades C1 and C2. 20

References 21

Supplementary methods

Sample processing and nucleic acid extraction

For stool specimens from Cyprus, Spain, Poland, and France, approximately 200 mg of material was suspended in 2 mL of an antibiotic solution containing penicillin (10 U/mL), streptomycin (10 mg/mL), and amphotericin B (5 mg/mL), together with 2 mL of pure chloroform. The suspension was vortexed for 2 min and centrifuged at 5,000× *g* for 15 min. A 200 µL aliquot of the aqueous phase was collected for nucleic acid extraction. The other clinical specimens were processed directly.

Total nucleic acids were extracted from 200 µL of sample using the *Maxwell* automated platform and the *RSC Blood DNA Kit* (Promega, Madison, WI, USA), eluted in 40 µL of nuclease-free water. For reverse transcription, 5.5 µL of nucleic acids was incubated with 0.5 µL of 2 µM reverse primer polyT3NC-HEVA01 (TTTTTTTTTTTTTTTTTTTTTTTTGCTATTCTGGTTATAACAAATTTACC) and 0.5 µL of 10 mM dNTP mix at 65 °C for 5 min to facilitate primer annealing. Reverse-transcription reactions (10 µL total volume) contained 0.5 µL of *SuperScript IV Reverse Transcriptase* (200 U/µL; Invitrogen, Thermo Fisher Scientific, Waltham, MA, USA), 0.1 µL of 0.1 M DTT, 2 µL of 5× First-Strand Buffer, and 0.5 µL of *RNaseOUT* (40 U/µL). Reactions were incubated at 55 °C for 30 min and inactivated at 80 °C for 10 min. The obtained cDNA was stored at −20 °C until further use.

PCR amplification of complete genomes

Full-length viral genomes were amplified by polymerase chain reaction (PCR) using *Platinum SuperFi2 DNA Polymerase* (Invitrogen, Thermo Fisher Scientific, Waltham, MA, USA) according to the manufacturer's instructions. Each 20 µL reaction contained 2 µL of cDNA, 10 µL of 2× reaction buffer, 6 µL of nuclease-free water, and 1 µL each of forward IFT7R5NC and reverse HEVA3NC primers (10 µM; GACAGCTTATCATCGTAATACGACTCACTATAGGGTTAAAACAGCCTGTGGGTTG and GCTATTCTGGTTATAACAAATTTACCCCCAC respectively). Thermal cycling was performed with initial denaturation at 98 °C for 1 min; 41 cycles of 98 °C for 5 s, 60 °C for 10 s, and 72 °C for 4 min 30 s; followed by a final extension at 72 °C for 5 min. Amplicon integrity (≈ 7.5 kb) was confirmed by electrophoresis on a 0.7% agarose gel.

Sequencing protocol of the Clermont-Ferrand laboratory (France)

Oxford Nanopore sequencing. Barcoded amplicons were pooled at equimolar concentrations, and library concentration was quantified fluorometrically using the *Qubit High-Sensitivity dsDNA Kit* (Thermo Fisher Scientific, Waltham, MA, USA). Following flow cell quality control, 11 µL of the 1,000 ng pooled library was loaded onto a *MinION Mk1C* sequencer using the *Rapid Barcoding Kit* (SQK-RBK110.96; Oxford Nanopore Technologies, Oxford, UK). Real-time basecalling and demultiplexing retained reads with quality scores > 8 and length > 1 kb with *MinKNOW* (*Oxford Nanopore Technologies Ltd., v24.11.8*). Adapter sequences were trimmed with *Porechop (v0.2.4, Wick RR, https://github.com/rrwick/Porechop)*, and reads were filtered using *Filtlong* (*v0.2.1, Wick RR, https://github.com/rrwick/Filtlong*). Reads meeting thresholds (Q > 8; length: 4,000–7,700 nt) were assembled de novo using *Flye v2.9.2* (Kolmogorov et al., 2019) and polished with *Medaka* (*v1.8.0, Oxford Nanopore Technologies Ltd., https://github.com/nanoporetech/medaka*). Genomes with a minimum coverage depth of 300× were retained for downstream analyses.

Illumina sequencing. Libraries were prepared using the *Illumina DNA Prep (M) Tagmentation Kit* (Illumina, San Diego, CA, États-Unis) following the manufacturer's protocol. Amplicons were enzymatically fragmented, normalized, and barcoded. Libraries were quantified with *Qubit*, diluted to 4 nM and further to 1.3 pM, then sequenced on a *NextSeq 500* platform (Clermont-Ferrand University Hospital Sequencing Facility) with a MidOutput flow cell (2 × 75 bp). Reads were demultiplexed with *bcl2fastq* v2.20.0.422, assessed with *FastQC* v0.11.9, and trimmed using *Trimmomatic* (Bolger et al., 2014). Contigs were assembled with *SPAdes* v3.15.3 (Bankevich et al., 2012), and filtered reads were mapped to consensus genomes using *bwa-mem* v0.7.17 (Li, 2013). Variant calling and intra-sample diversity analyses were performed using *GATK* v4.2.5.0 (McKenna et al., 2010) and *BCFtools* v1.10.2 (Danecek et al., 2021).

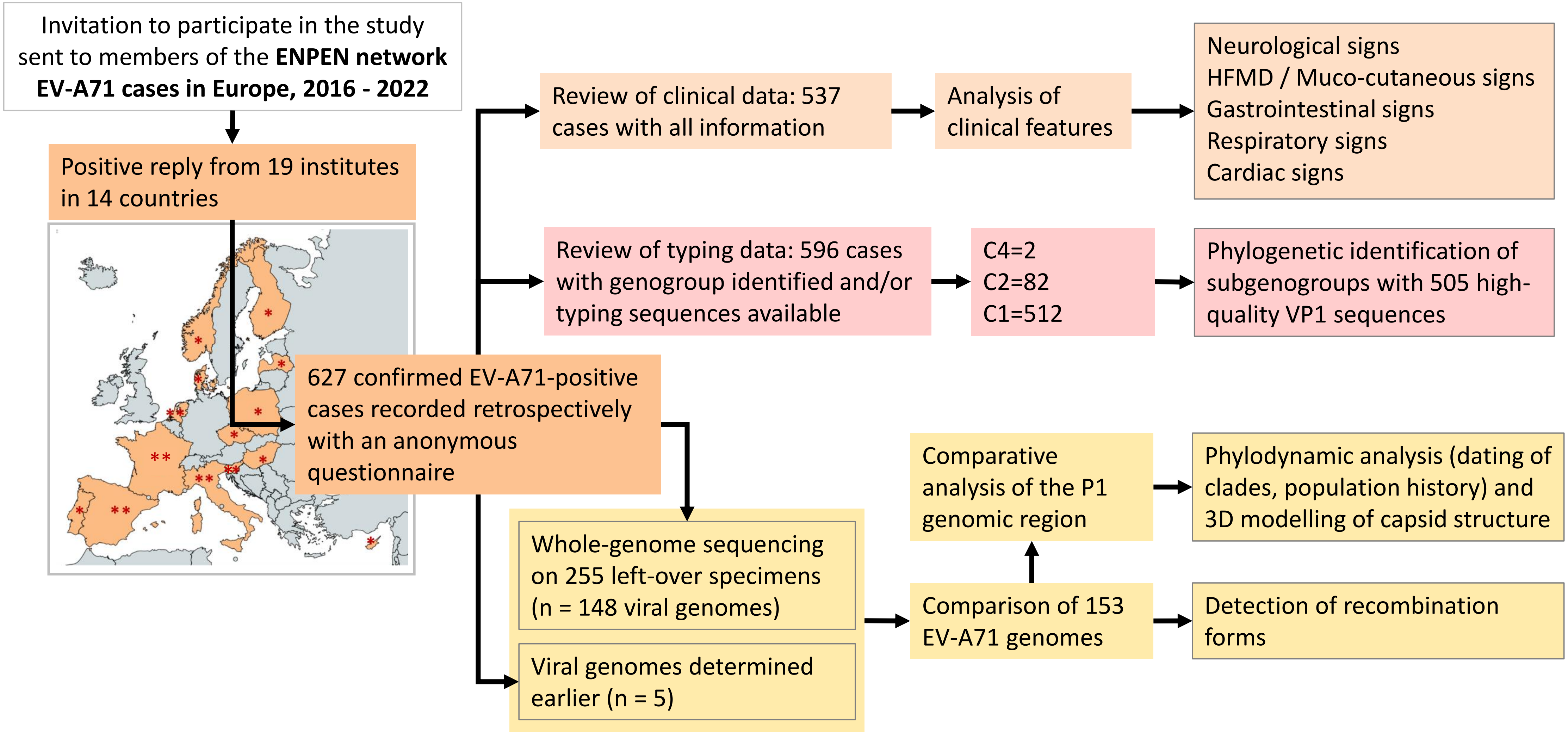

Figure S1. Complete workflow of data analyses performed in the study.

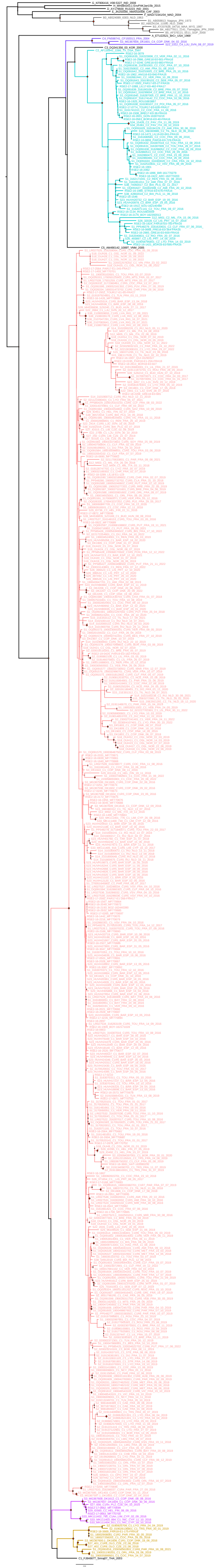

Figure S2. Phylogenetic clustering of EV-A71 VP1 sequences and assignment to extant genogroups.

This phylogenetic tree corresponds to the collapsed tree shown in Fig. 3. It was reconstructed from 437 VP1 sequences using a maximum likelihood method. Sequences from the present study were coloured according to their assignment to genogroups and subgenogroups (see colour code below) for clarity. The size and colour of the circles located at the nodes are proportional to the strength of the SH-like support test: the larger and redder the circle, the stronger the support. A single reference sequence was used for genogroups A, D, E, F, G, and H, as well as for subgenogroups C1 to C5 and B0 to B5. In the tree, the reference sequences of genogroups A, D, E, F, G, and H, as well as those of subgenogroups C1, C3, and C5, are shown in black, whereas those of subgenogroup B appear in grey. Sequences assigned to subgenogroup C4 are shown in purple and those of C2 in green. Finally, sequences belonging to the clades of subgenogroup C1 are coloured pastel pink for C1.1, orange for C1.2, and pink for C1.3. The sequences analysed in this study were geographically distributed as follows: Seven sequences in subclade C1.1: Cyprus (n=3), Denmark (n=2), the Czech Republic (n=1), and Finland (n=1). Ten sequences in subclades C1.2: France (n=7), the Czech Republic (n=2), and Denmark (n=1). Three Hundred sixty-two sequences subclade C1.3 (95% of the C1 sequences) : France (n=212, 59% of sequences), Spain (n=50, 14% of sequences), Netherlands (n=21), Norway (n=20), Denmark (n=19), Portugal (n=7), Slovenia (n=6), Finland (n=5), Italy, (n=5), Latvia (n=5), Hungary (n=3), Cyprus (n=3), Czech Republic (n=3), Poland (n=3). Subclade C1.3 includes widespread viruses that have been circulating in Europe since 2015 and were initially detected in Germany. Overall, the two C1.2 and C1.3 subclades accounted for 98% (n=372/379) of sequences assigned to EV-A71 subgenogroup C1. The C1.2 and C1.3 sequences were formerly designated C1-like (Midgley et al., 2017). Thirty-nine sequences in subgenogroup C2: France (n=29), Italy (n=3), Netherlands (n=1), Poland (n=2), Portugal (n=2), and Spain (n=2); And two sequences in subgenogroup C4: Denmark (n=1), and Slovenia (n=1).

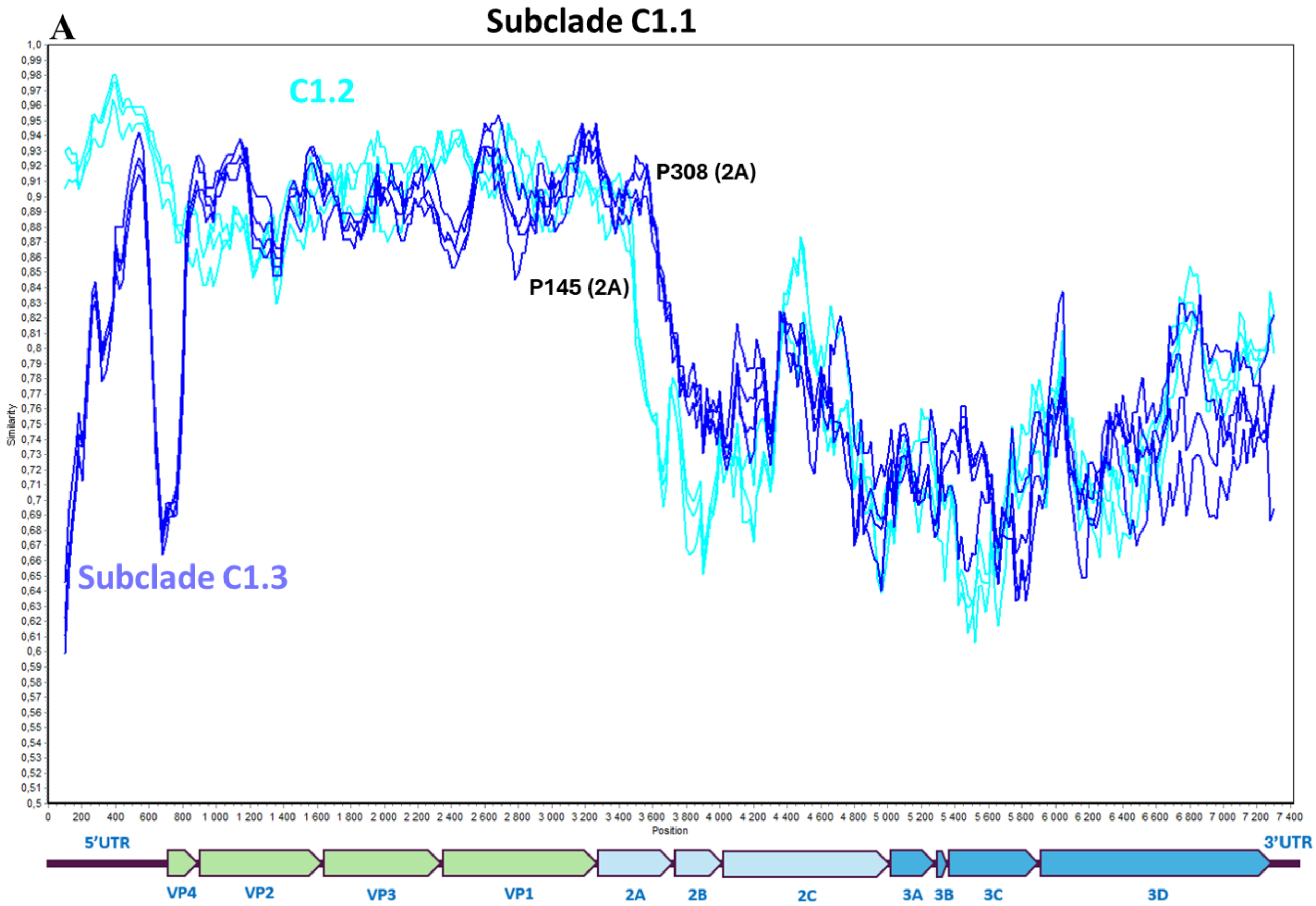
A
Subclade C1.1
C1.2
P308 (2A)
P145 (2A)
Subclade C1.3
Similarity
Position
5'UTR
3'UTR
VP4
VP2
VP3
VP1
2A
2B
2C
3A
3B
3C
3D

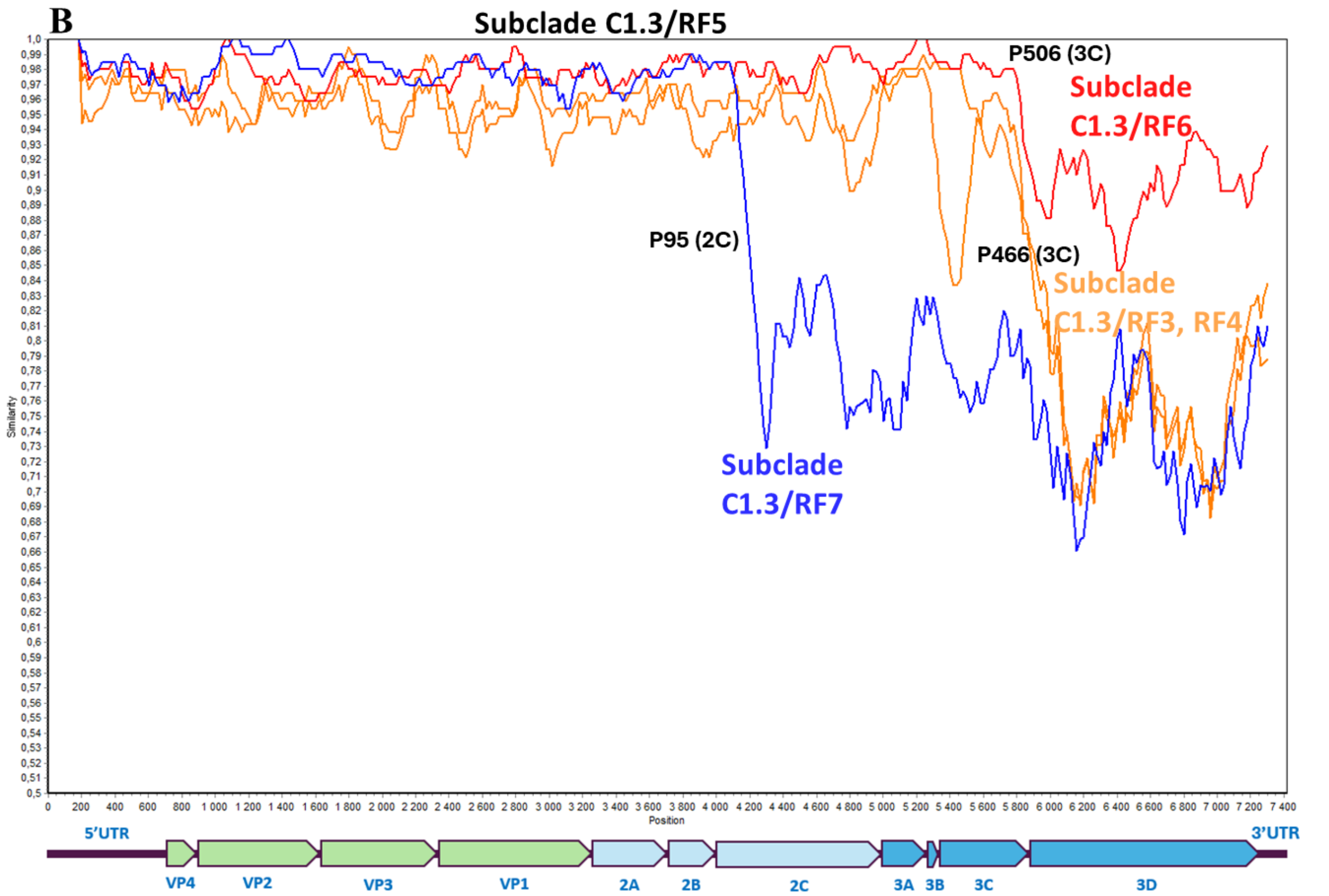
B
Subclade C1.3/RF5
P506 (3C)
Subclade
C1.3/RF6
P95 (2C)
P466 (3C)
Subclade
C1.3/RF3, RF4
Subclade
C1.3/RF7
Similarity
Position
5'UTR
3'UTR
VP4
VP2
VP3
VP1
2A
2B
2C
3A
3B
3C
3D

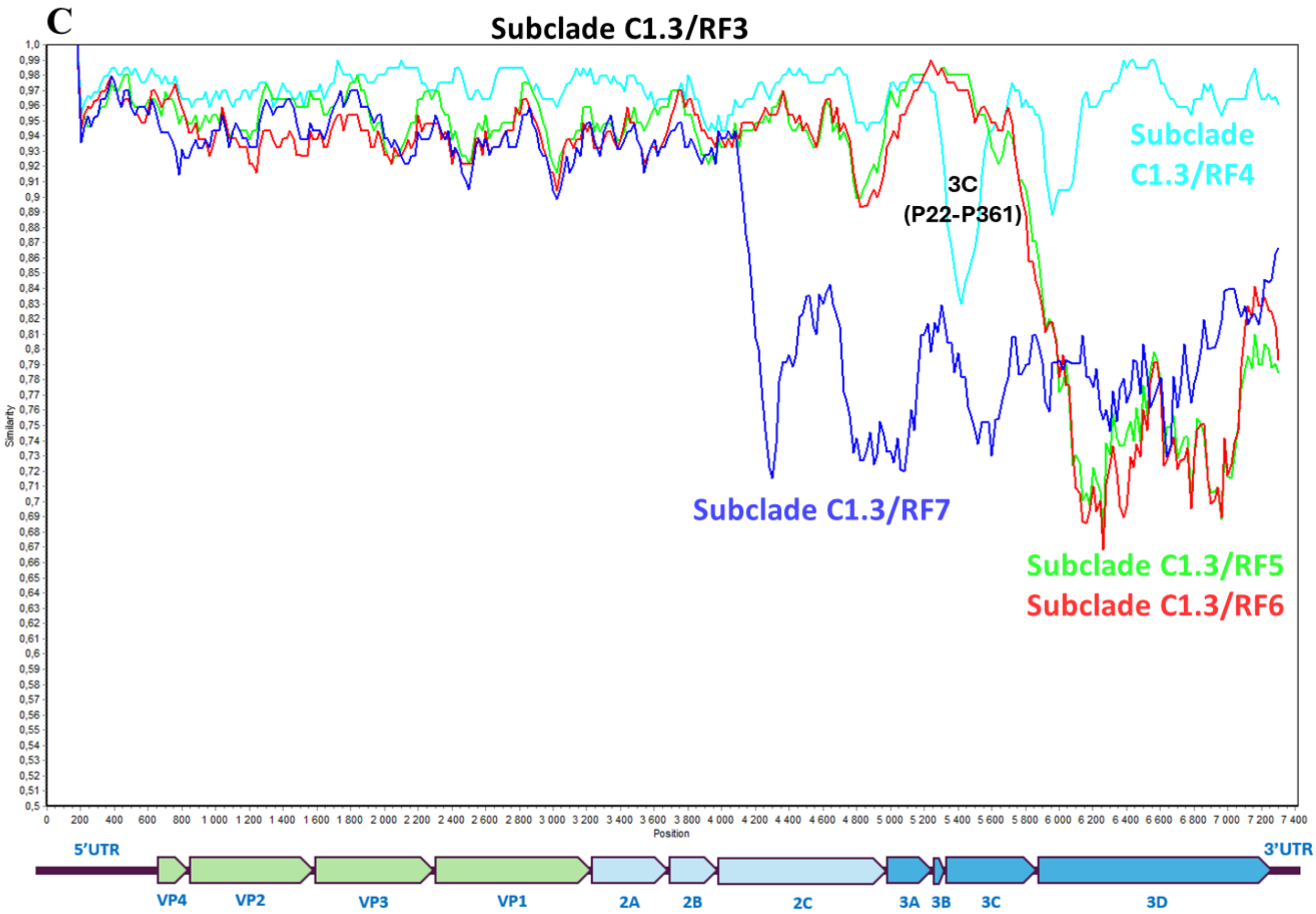
C
Subclade C1.3/RF3
Subclade C1.3/RF4
3C
(P22-P361)
Subclade C1.3/RF7
Subclade C1.3/RF5
Subclade C1.3/RF6
Similarity
Position
5'UTR
3'UTR
VP4
VP2
VP3
VP1
2A
2B
2C
3A
3B
3C
3D

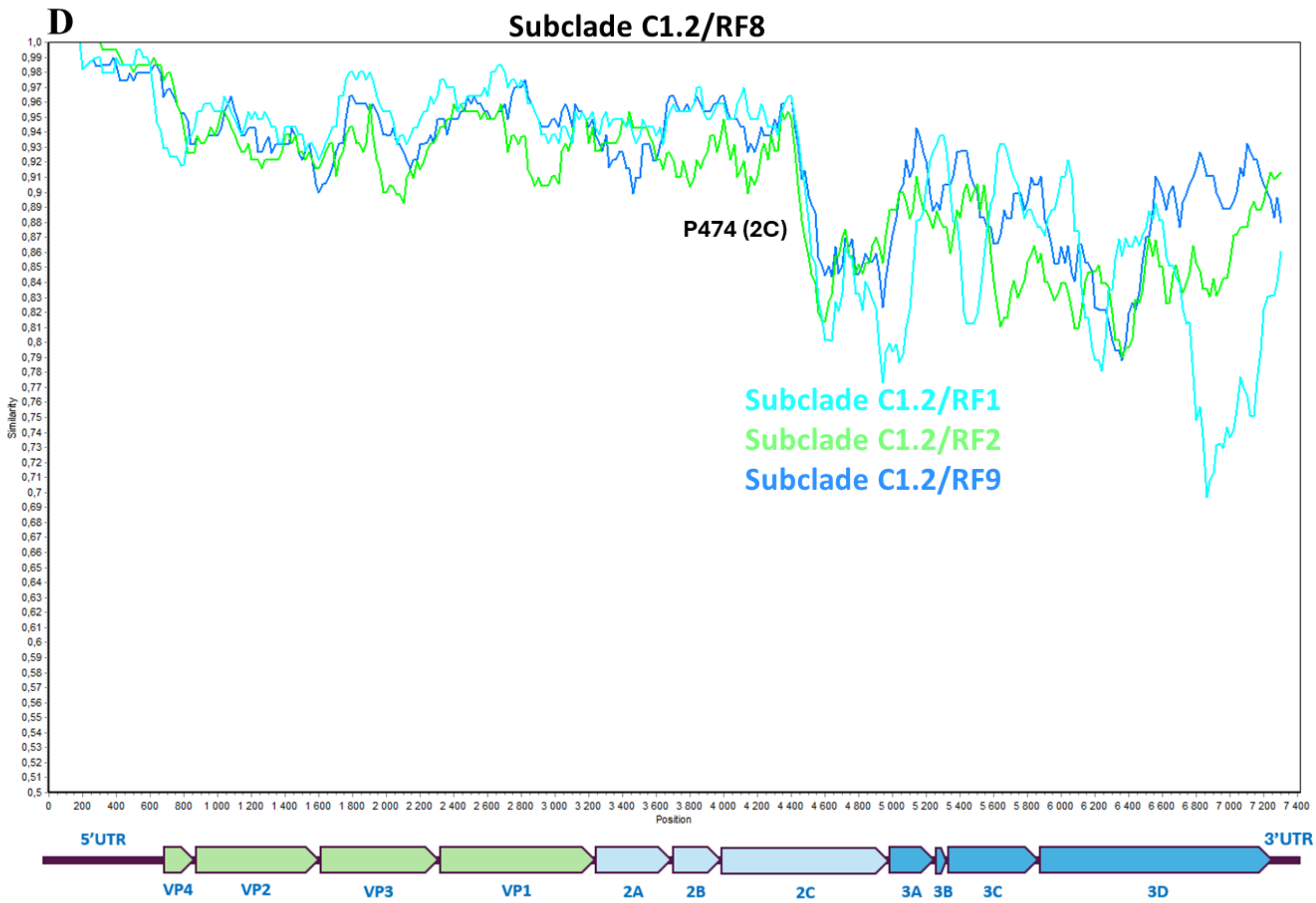
D
Subclade C1.2/RF8
P474 (2C)
Subclade C1.2/RF1
Subclade C1.2/RF2
Subclade C1.2/RF9
Similarity
Position
5'UTR
3'UTR
VP4
VP2
VP3
VP1
2A
2B
2C
3A
3B
3C
3D

E

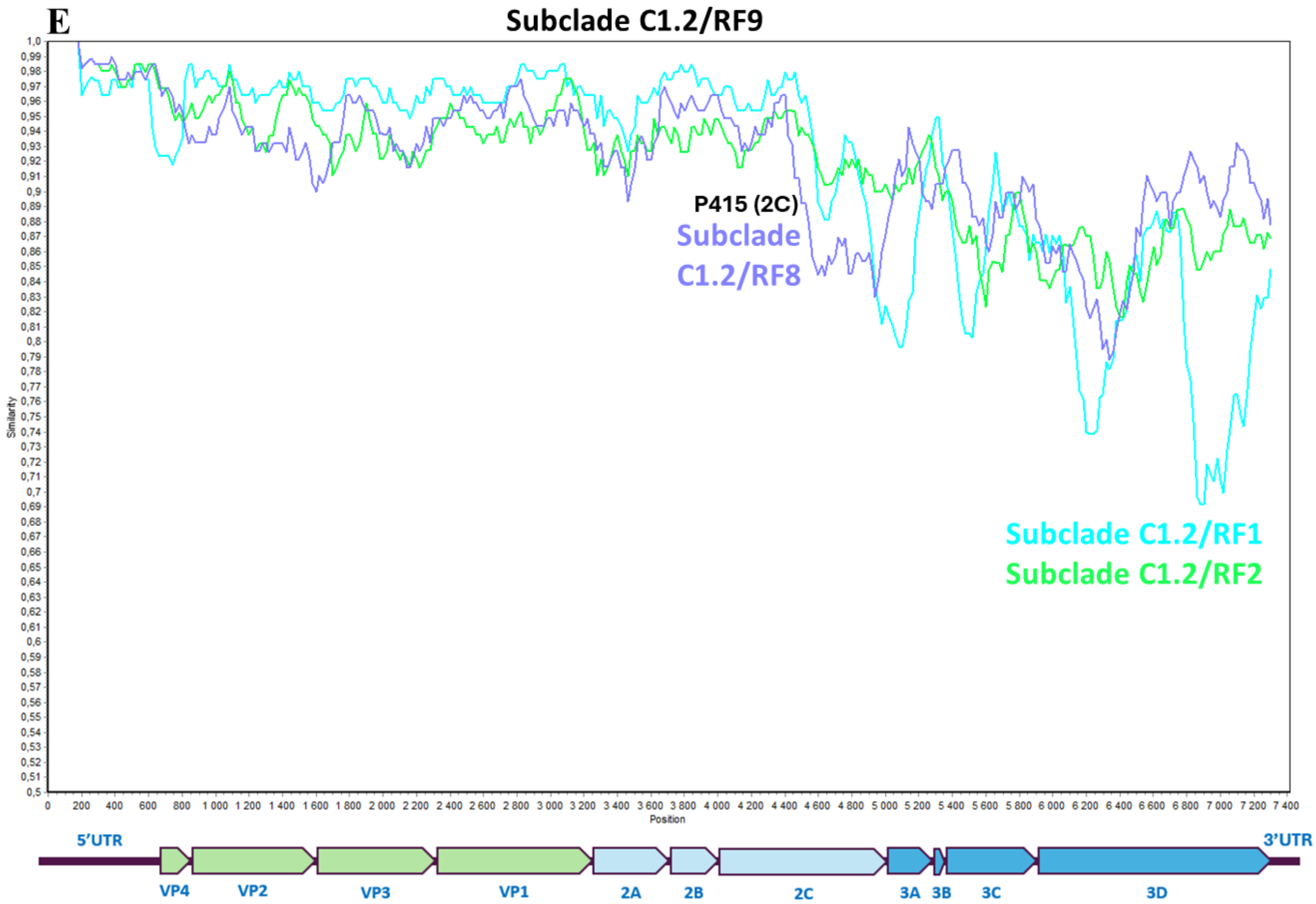
Subclade C1.2/RF9
Similarity
Position
P415 (2C)
Subclade C1.2/RF8
Subclade C1.2/RF1
Subclade C1.2/RF2
5'UTR
3'UTR
VP4
VP2
VP3
VP1
2A
2B
2C
3A
3B
3C
3D

F

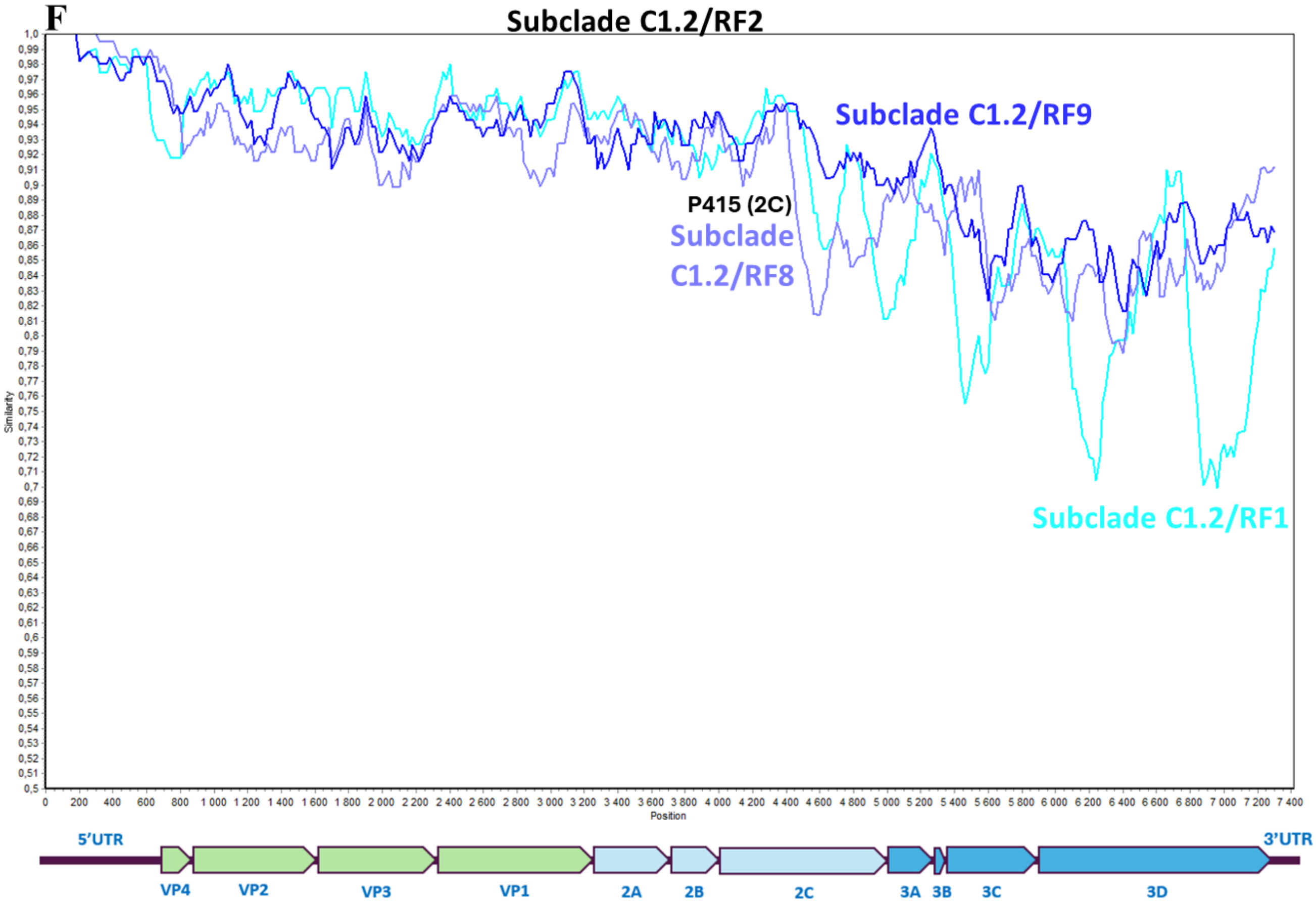
Subclade C1.2/RF2
Similarity
Position
Subclade C1.2/RF9
P415 (2C)
Subclade C1.2/RF8
Subclade C1.2/RF1
5'UTR
3'UTR
VP4
VP2
VP3
VP1
2A
2B
2C
3A
3B
3C
3D

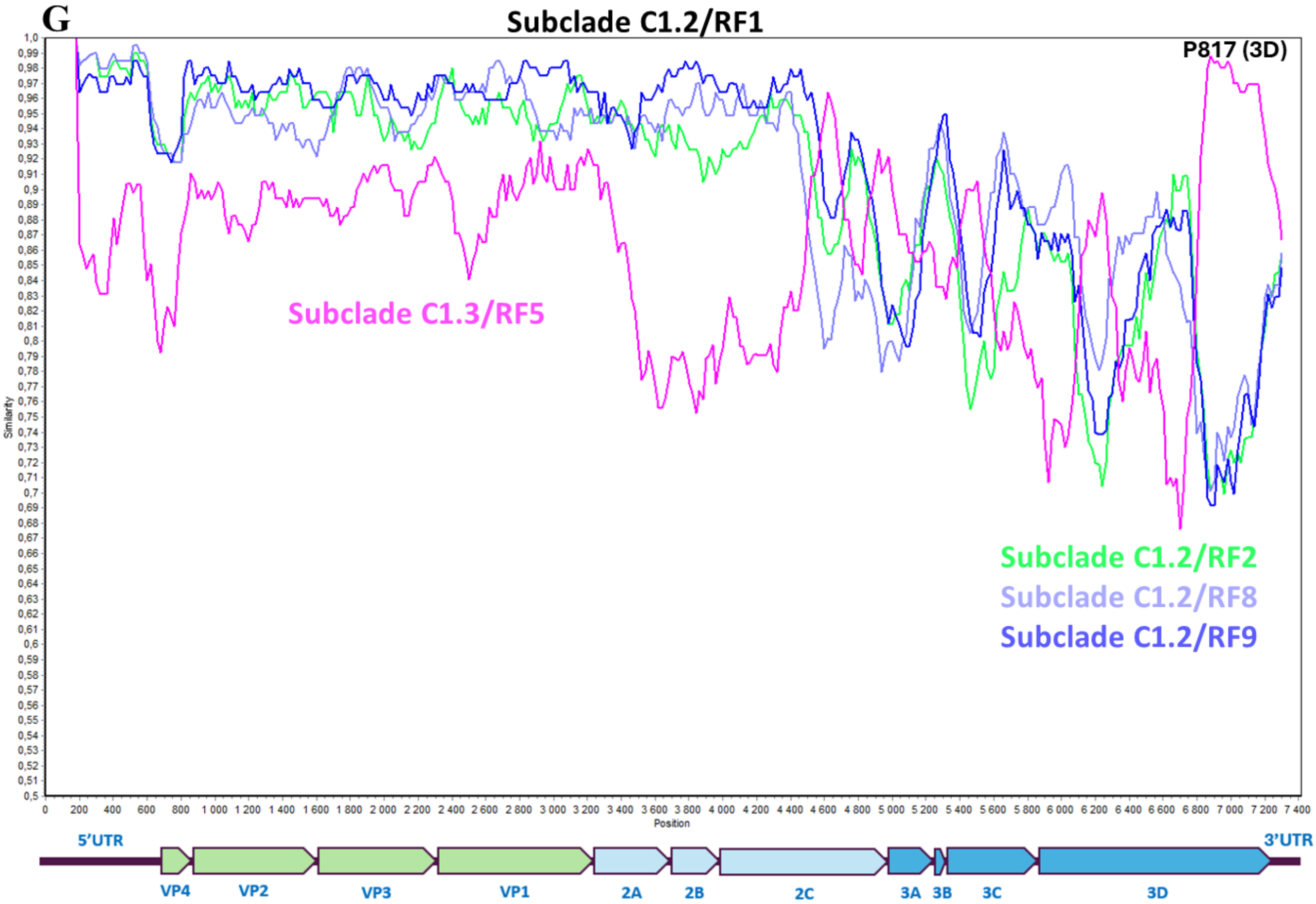

Figure S3. Nucleotide similarity patterns of complete EV-A71 genomes determined with Simplot and assignment to recombinant forms (RF).

For each RF, a representative reference sequence was selected and used for alignment and similarity analysis using SimPlot (Lole et al., 1999) . Panel (A) shows the analysis across subclades C1.1, C1.2, and C1.3. The same approach was applied within subclade C1.3 (panels B–C) and within subclade C1.2 (panels D–G) to show genetic difference between each RFs. The reference sequence used in each analysis is indicated in the title of the corresponding panel.

Analysis of relationships between EV-A71 RFs.

Fs 1, 2, 8, and 9 shared a common P2 genomic region distinct from that of the C1.1 subclade. Recombination breakpoints within the 2C gene sequence gave rise to divergent P3 regions in each of these four RFs. The other five RFs (RF3 to RF7) shared a common 5'NCR and common 2A and 2B gene sequences, and thus were divergent from the C1.1 subclade and from RFs 1, 2, 8 and 9. RFs 3, 4, 6 and 7 underwent distinct recombination events within the P2 region (in RF7) and the P3 region (in RFs 3, 4 and 6), resulting in their divergence from RF5. Furthermore, the high nucleotide similarity (95%) at the 3′ end of RF1 and RF5 (from position 817 of the 3D gene) supports the occurrence of direct or indirect genomic exchange between these two RFs.

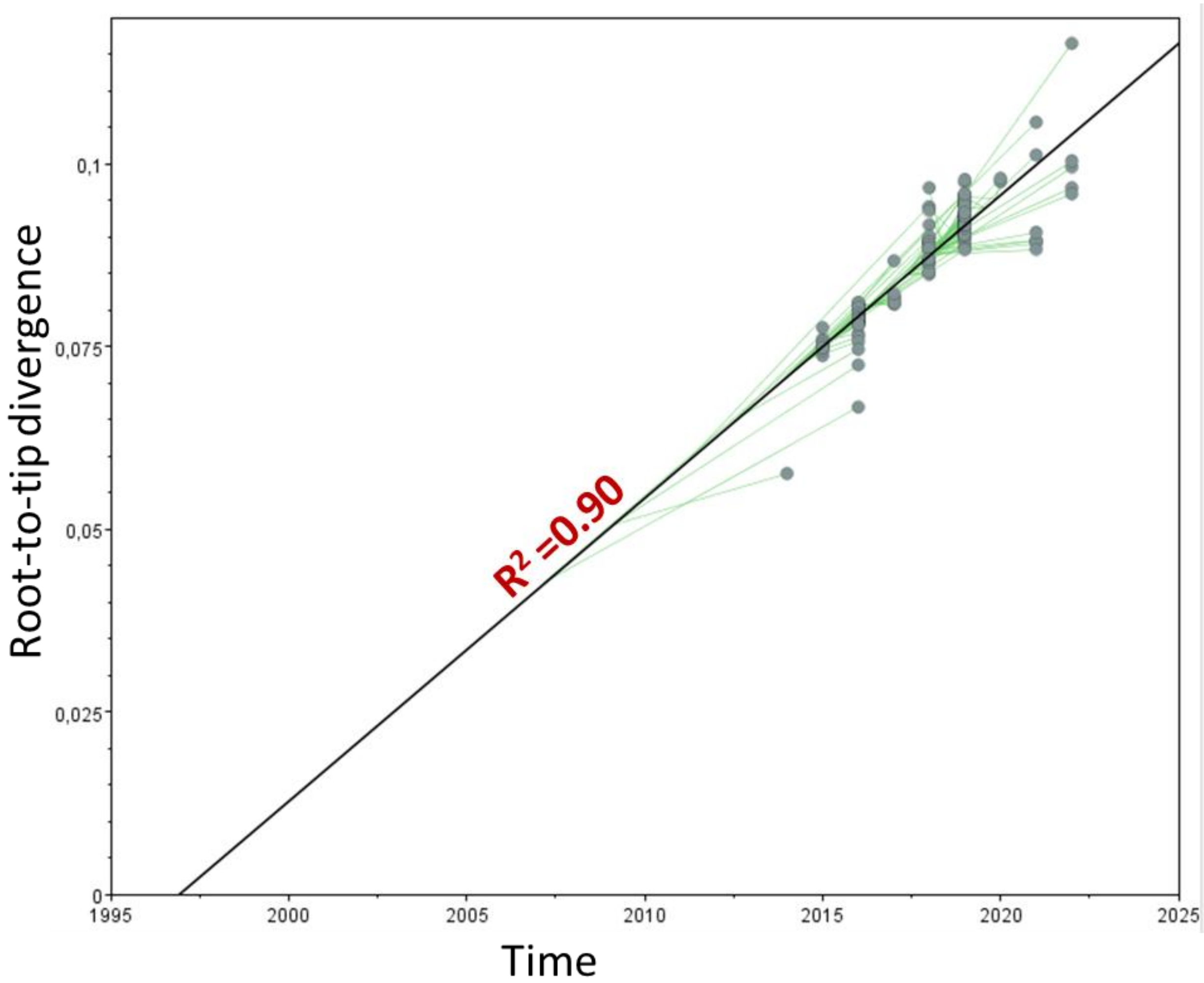

Figure S4. Assessment of the molecular clock of the P1 genomic region of EV-A71.
The robustness of the relationship between root-to-tip genetic distances and time was assessed using the correlation coefficient (R^2). With an estimated R^2 value of 0.90, these results highlight a strong temporal signal in the P1 genomic region of EV-A71, suggesting that the nucleotide sequences can be used for reconstructing the genealogy of viral lineages.

Owing to the large number of recombination breakpoints in the non-structural genomic regions of EV-A71, the evolutionary history of C1 viruses was based on the P1 capsid region, which is non- or poorly recombining in EV genomes (Nikolaidis et al., 2019). Molecular clock analysis showed a strong temporal signal in the P1 sequences ($R^2 = 0.9$; Supplementary Fig. S4), supporting the robustness of subsequent Bayesian time-scaled phylogenetic inference.

Molecular clock analyses suggested that subclades C1.2 and C1.3 emerged between 2009 and 2014, with estimated evolutionary rates of 5.15×10^{-3} and 4.80×10^{-3} nucleotide substitutions per site per year, respectively. Within C1.3, RF3 and RF4 evolved at an estimated rate of 3.95×10^{-3} nucleotide substitutions per site per year, whereas RF5, RF6, and RF7 had a higher rate of 5.68×10^{-3} nucleotide substitutions per site per year, a value comparable to that inferred for the C1.1 subclade (5.29×10^{-3} nucleotide substitutions per site per year). The differences in the evolutionary rates between subclades C1.1, C1.2, and C1.3 (5.29×10^{-3}, 5.15×10^{-3}, and 4.80×10^{-3} substitutions/site/year, respectively) are not significant because their corresponding 95% highest posterior density intervals overlap. The similar evolutionary rates estimated for C1.1, C1.2, and C1.3 suggest comparable long-term evolutionary dynamics, and the observed differences should not be interpreted as evidence of differential epidemiological fitness without additional phenotypic or epidemiological data.

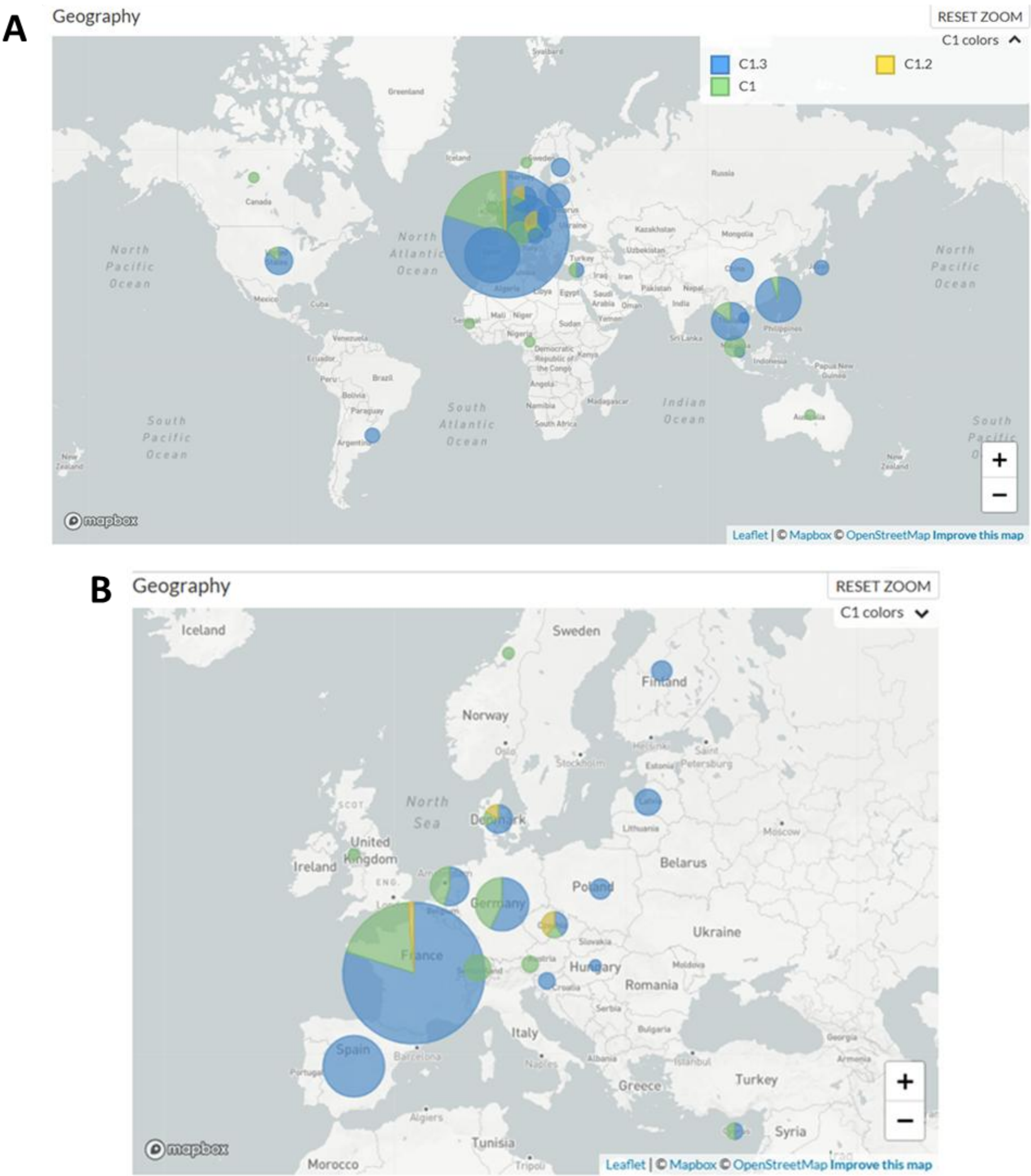

Figure S5. Visualisation of the geographic distribution of EV-A71 C1 subclades using Nextstrain.
The genome sequences from this study were compared with all EV-A71 genomes publicly available from GenBank to generate a dataset of 1,975 P1 sequences. The geographic distribution of the subset of C1 sequences is shown here, comprising 303 sequences sampled between 1991 and 2024, of which 149 are from our study. The worldwide spatial distribution of C1 subclades is visualised in (A), with (B) showing their distribution specifically in European countries.

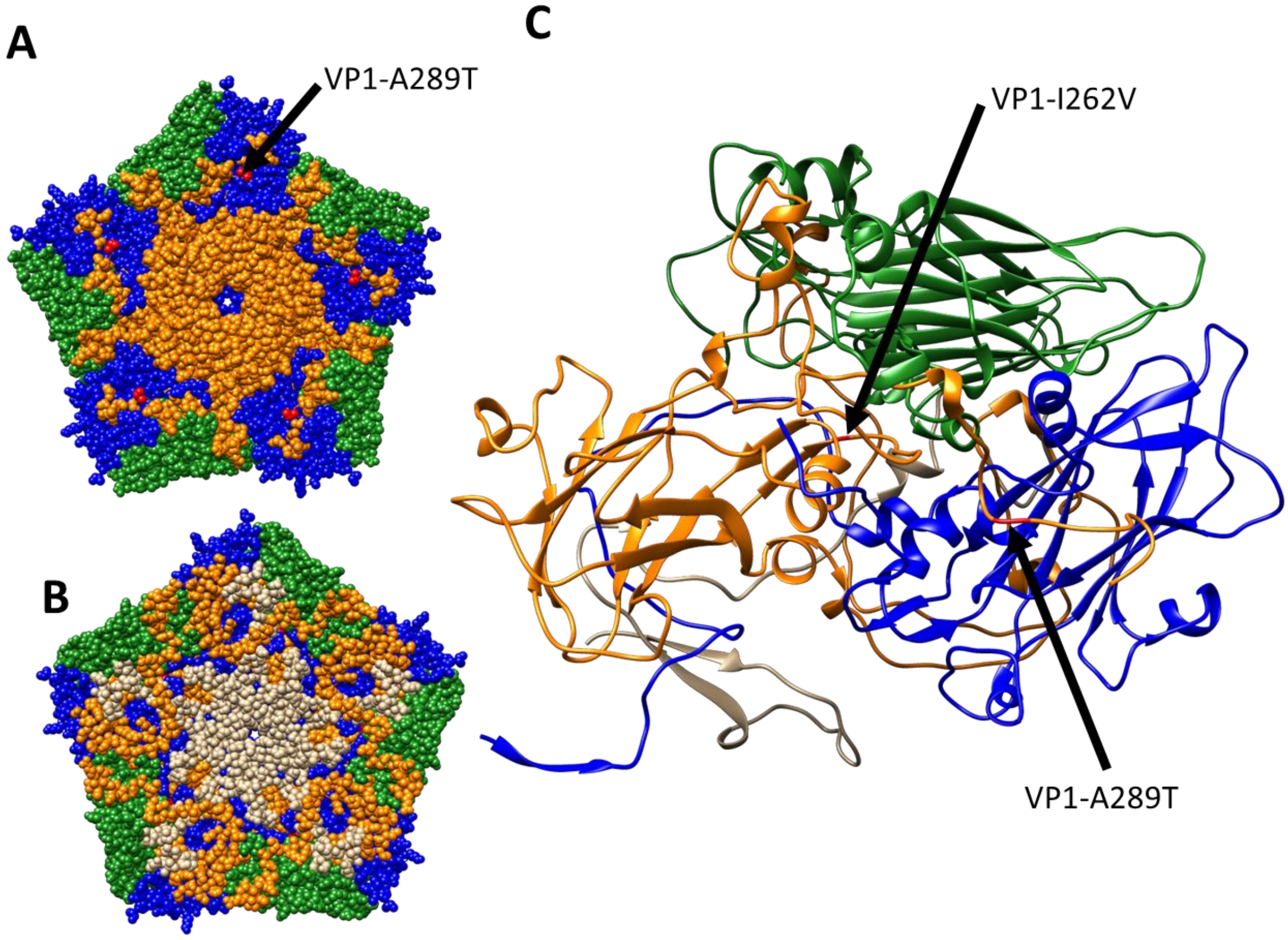

Figure S6. Localisation of the two common mutations on all C1 lineages (VP1: 262 and 289) on the structural modeling of the EV-A71 capsid pentamer

Reference sequences LR027531 was used to generate capsid pentamer models using AlphaFold. The predicted models were validated by comparison with the available crystal structure (PDB ID: 8E2X; strain 4643; Catching *et al.* 2023). Structural representations were generated using UCSF Chimera (Pettersen et al., 2004). Capsid proteins are shown in different colours: VP1 (orange), VP2 (green), VP3 (blue), and VP4 (grey). Amino acid substitutions common on all C1 strains, and localised near the base of the phylogenetic tree of Fig. 6A inferred by Nextstrain are highlighted in red. Panels A and B correspond to respectively external and internal views of the capsid pentamer. Panel C present enlarged views highlighting the spatial proximity of mutations within the capsid structure and their localization within β-sheets and α-helices.

Table S1. GenBank accession numbers of complete genomes used in this study.

Number	Sequence designation	Accession number	Subclade	Country [a]	Sampling date	Length	Reference
1	3171840071	PP548278	C1 RF5	FRA	22/03/2017	7373	This study
2	3163020011	LR027546	C1 RF5	FRA	20/10/2016	7411	This study
3	316302002	LR027531	C1 RF5	FRA	18/10/2016	7409	This study
4	316250102	LR027524	C1 RF5	FRA	30/08/2016	7409	This study
5	HUVH75020	PQ284431	C1 RF5	ESP	25/09/2016	6910	This study
6	HUVH42668	PQ284432	C1 RF5	ESP	28/06/2016	7145	This study
7	HUVH16244	PQ284434	C1 RF5	ESP	11/05/2016	7383	This study
8	HUVH24479	PQ284435	C1 RF5	ESP	25/05/2016	7280	This study
9	HUVH19873	PQ284433	C1 RF5	ESP	18/05/2016	6817	This study
10	HUVH19320	PQ284438	C1 RF5	ESP	18/05/2016	6569	This study
11	HUVH16309	PQ284448	C1 RF5	ESP	11/05/2016	7107	This study
12	3162860301	OQ091594	C1 RF5	FRA	04/10/2016	7391	This study
13	316286036	LR027538	C1 RF5	FRA	04/10/2016	7411	This study
14	316286034	LR027537	C1 RF5	FRA	04/10/2016	7411	This study
15	316286032	LR027536	C1 RF5	FRA	04/10/2016	7411	This study
16	HUVH24826	PQ284436	C1 RF5	ESP	26/05/2016	7257	This study
17	HUVH27800	PQ284437	C1 RF5	ESP	31/05/2016	7379	This study
18	3170520021	OQ091593	C1 RF5	FRA	21/02/2017	7391	This study
19	316307017	LR027522	C1 RF5	FRA	10/08/2016	7411	This study
20	316307036	LR027532	C1 RF5	FRA	11/10/2016	7411	This study
21	316307016	LR027521	C1 RF5	FRA	10/08/2016	7411	This study
22	316263109	LR027534	C1 RF5	FRA	14/09/2016	7412	This study
23	HUVH24346	PQ284442	C1 RF5	ESP	26/05/2016	7335	This study
24	HUVH20361	PQ284443	C1 RF5	ESP	18/05/2016	7393	This study
25	HUVH16864	PQ284444	C1 RF5	ESP	12/05/2016	7332	This study
26	316180009	LR027529	C1 RF5	FRA	24/06/2016	7411	This study
27	HUVH33719	PQ284439	C1 RF5	ESP	10/06/2016	7302	This study
28	HUVH20311	PQ284440	C1 RF5	ESP	18/05/2016	7259	This study
29	HUVH21667	PQ284441	C1 RF5	ESP	20/05/2016	7392	This study
30	HUVH18902	PQ284445	C1 RF5	ESP	13/05/2016	7375	This study
31	HUVH16005	PQ284446	C1 RF5	ESP	10/05/2016	7376	This study
32	HUVH27854	PQ284447	C1 RF5	ESP	01/06/2016	7368	This study
33	316148153	LR027527	C1 RF5	FRA	03/05/2016	7411	This study
34	190025277202	OQ091586	C1 RF5	FRA	22/05/2019	7392	This study
35	190027101601	OQ091587	C1 RF5	FRA	04/06/2019	7393	This study
36	190032189602	OQ091590	C1 RF5	FRA	02/07/2019	7392	This study
37	180052732702	PP548280	C1 RF5	FRA	31/10/2018	7338	This study
38	190050240602	OQ091589	C1 RF5	FRA	22/10/2019	7391	This study
39	190033801602	OQ091588	C1 RF5	FRA	09/07/2019	7381	This study
40	3170060071	OQ091591	C1 RF5	FRA	05/12/2016	7391	This study
41	170043237202	OQ026335	C1 RF5	FRA	29/05/2017	7410	This study
42	316238077	LR027528	C1 RF5	FRA	11/08/2016	7411	This study
43	190052161302	OQ091596	C1 RF5	FRA	27/05/2019	7390	This study

44	3172360461	OQ026332	C1 RF5	FRA	13/07/2017	7410	This study
45	170050125902	OQ026333	C1 RF5	FRA	03/07/2017	7410	This study
46	17501259	LR027539	C1 RF5	FRA	03/07/2017	7413	This study
47	180031473702	OQ026334	C1 RF5	FRA	29/06/2018	7408	This study
48	316259064	LR027525	C1 RF5	FRA	05/09/2016	7412	This study
49	3101800648	PQ284454	C1 RF5	NLD	05/11/2018	7313	This study
50	3101800675	PQ284455	C1 RF5	NLD	16/11/2018	7314	This study
51	2109059655	PQ284497	C1 RF5	LVA	17/09/2021	7381	This study
52	2108079811	PQ284498	C1 RF5	LVA	10/09/2021	7296	This study
53	2107047291	PQ284496	C1 RF5	LVA	16/07/2021	7399	This study
54	2108039176	PQ284499	C1 RF5	LVA	13/08/2021	7385	This study
55	2107083641	PQ284500	C1 RF5	LVA	29/07/2021	7309	This study
56	HUVH25514	PQ284449	C1 RF5	ESP	13/04/2018	7374	This study
57	916	PQ284476	C1 RF5	CZE	16/10/2017	6869	This study
58	292	PQ284501	C1 RF5	CZE	22/07/2019	7386	This study
59	190033373602	OQ091583	C1 RF5	FRA	25/06/2019	7390	This study
60	190035098601	OQ091584	C1 RF5	FRA	03/06/2019	7391	This study
61	61502019	PQ284484	C1 RF5	POL	03/07/2019	7390	This study
62	220014315103	PP585429	C1 RF5	FRA	08/03/2022	7388	This study
63	HUVH80884	PQ284450	C1 RF5	ESP	01/11/2019	7348	This study
64	190031430703	OQ091580	C1 RF5	FRA	01/07/2018	7389	This study
65	190036487502	OQ091579	C1 RF5	FRA	03/08/2019	7389	This study
66	180021297002	OQ091581	C1 RF5	FRA	25/04/2018	7390	This study
67	180054825602	OQ091585	C1 RF5	FRA	15/11/2018	7389	This study
68	36572018	PQ284483	C1 RF5	POL	16/05/2018	7390	This study
69	190023000201	OQ091573	C1 RF5	FRA	09/05/2019	7391	This study
70	190050742201	OQ091574	C1 RF5	FRA	27/10/2019	7359	This study
71	190027099802	OQ091578	C1 RF5	FRA	03/06/2019	7389	This study
72	190032996701	OQ091576	C1 RF5	FRA	10/07/2019	7389	This study
73	190031812802	OQ091575	C1 RF5	FRA	05/07/2019	7404	This study
74	190033738402	OQ091577	C1 RF5	FRA	12/07/2019	7390	This study
75	3101900663	PQ284453	C1 RF5	NLD	22/10/2019	7383	This study
76	3102000132	PQ284457	C1 RF5	NLD	30/03/2020	7380	This study
77	3101900756	PQ284458	C1 RF5	NLD	29/11/2019	7298	This study
78	190043354802	OQ091582	C1 RF5	FRA	10/09/2019	7389	This study
79	220063498502	PP585427	C1 RF5	FRA	21/11/2022	7389	This study
80	220068170502	PP585428	C1 RF5	FRA	12/12/2022	7388	This study
81	220056349502	PP585426	C1 RF5	FRA	17/10/2022	7391	This study
82	210068108802	OQ091597	C1 RF5	FRA	19/11/2021	7393	This study
83	3162501011	LR027523	C1 RF5	FRA	30/08/2016	4590	This study
84	3170520261	PP548276	C1 RF5	FRA	14/12/2017	7109	This study
85	3163070231	LR027526	C1 RF5	FRA	07/09/2016	5047	This study
86	3101900712	PQ284456	C1 RF5	NLD	12/11/2019	7302	This study
87	2414	PQ284490	C1 RF5	SVN	16/05/2019	7114	This study
88	HUVH91612	PQ284429	C1 RF6	ESP	25/02/2019	7400	This study
89	190031370202	OQ091599	C1 RF6	FRA	01/07/2019	7392	This study

90	190057539001	OQ091600	C1 RF6	FRA	21/09/2019	7391	This study
91	190031161302	OQ091608	C1 RF6	FRA	26/06/2019	7391	This study
92	190042783001	OQ091609	C1 RF6	FRA	02/09/2019	7391	This study
93	180054826202	OQ026325	C1 RF6	FRA	19/11/2018	7410	This study
94	180054043101	OQ026326	C1 RF6	FRA	09/10/2018	7410	This study
95	180023322702	OQ026328	C1 RF6	FRA	23/03/2018	7410	This study
96	180023322602	OQ026327	C1 RF6	FRA	14/05/2018	7409	This study
97	180027462502	OQ026331	C1 RF6	FRA	05/06/2018	7410	This study
98	180027462102	OQ026330	C1 RF6	FRA	05/06/2018	7410	This study
99	180027461802	OQ026329	C1 RF6	FRA	06/06/2018	7410	This study
100	180051161202	OQ026324	C1 RF6	FRA	15/10/2018	7410	This study
101	190034094301	OQ091603	C1 RF6	FRA	19/07/2019	7391	This study
102	190035104201	OQ091604	C1 RF6	FRA	10/07/2019	7391	This study
103	190035996201	OQ091601	C1 RF6	FRA	17/07/2019	7391	This study
104	190053814002	OQ091602	C1 RF6	FRA	06/11/2019	7391	This study
105	190033943602	OQ091610	C1 RF6	FRA	16/07/2019	7392	This study
106	54912019	PQ284485	C1 RF6	POL	12/06/2019	7392	This study
107	190047245702	OQ091606	C1 RF6	FRA	04/10/2019	7391	This study
108	190049867602	OQ091605	C1 RF6	FRA	07/10/2019	7391	This study
109	190026311702	OQ091598	C1 RF6	FRA	30/05/2019	7391	This study
110	190034916401	OQ091607	C1 RF6	FRA	19/07/2019	7390	This study
111	190038932901	OQ091611	C1 RF6	FRA	20/08/2019	7391	This study
112	190048543102	OQ091612	C1 RF6	FRA	15/10/2019	7390	This study
113	190056852201	OQ091613	C1 RF6	FRA	06/12/2019	7391	This study
114	190012246401	OQ091614	C1 RF6	FRA	05/03/2019	7391	This study
115	2035	PQ284489	C1 RF6	SVN	20/05/2019	7375	This study
116	220034522702	PP585425	C1 RF6	FRA	17/06/2022	7391	This study
117	190033383501	PP548277	C1 RF6	FRA	10/07/2019	6841	This study
118	316286037	LR027533	C1 RF4	FRA	27/09/2016	7412	This study
119	190033382701	OQ091615	C1 RF7	FRA	07/07/2019	7390	This study
120	795	PQ284475	C1	CYP	02/09/2016	7394	This study
121	656	PQ284504	C1	CZE	04/10/2016	7278	This study
122	401	PQ284502	C1 RF9	CZE	23/08/2018	7311	This study
123	402	PQ284503	C1 RF9	CZE	23/08/2018	7339	This study
124	210056561801	OQ091616	C1 RF8	FRA	16/08/2021	7389	This study
125	18002908081	PQ284300	C1 RF2	FRA	30/05/2018	7405	This study
126	316302008	OQ091627	C2	FRA	20/10/2016	7389	This study
127	42802018	PQ284487	C2	POL	11/06/2018	7389	This study
128	74092017	PQ284486	C2	POL	02/11/2017	7389	This study
129	3161620631	PP548279	C2	FRA	07/06/2016	7394	This study
130	316286052	OQ091630	C2	FRA	28/09/2016	7389	This study
131	316307009	OQ091631	C2	FRA	28/07/2016	7389	This study
132	316307018	OQ091632	C2	FRA	13/08/2016	7389	This study
133	316300020	OQ091633	C2	FRA	24/10/2016	7389	This study
134	316194129	OQ091629	C2	FRA	30/06/2016	7389	This study
135	316287005	OQ091640	C2	FRA	11/10/2016	7389	This study

136	316299004	OQ091641	C2	FRA	11/10/2016	7389	This study
137	316190108	OQ091639	C2	FRA	05/07/2016	7389	This study
138	316190107	OQ091638	C2	FRA	05/07/2016	7389	This study
139	316174142	OQ091637	C2	FRA	10/06/2016	7389	This study
140	316310001	OQ091642	C2	FRA	25/10/2016	7389	This study
141	316190103	OQ091643	C2	FRA	05/07/2016	7389	This study
142	316301003	OQ091636	C2	FRA	07/10/2016	7389	This study
143	316323006	OQ091635	C2	FRA	02/11/2016	7389	This study
144	HUVH25863	PQ284430	C2	ESP	28/05/2016	7087	This study
145	DK1605	MG367599	C1 RF5	DNK	02/06/2016	7152	Midgley et al., 2017
146	DK1604	MG367598	C1 RF5	DNK	23/05/2016	7152	Midgley et al., 2017
147	DK1602	MG367596	C1 RF5	DNK	29/02/2016	7152	Midgley et al., 2017
148	DK1606	MG367600	C1 RF1	DNK	18/06/2016	7140	Midgley et al., 2017
149	DK1403	MG367595	C1 RF3	DNK	01/11/2014	7142	Midgley et al., 2017
150	371654	PZ364747	C1 RF5	PRT	08/05/2017	7399	This study
151	397492	PZ364748	C1 RF5	PRT	26/10/2022	7399	This study
152	505621	PZ364750	C1 RF6	PRT	22/07/2019	7401	This study
153	507442	PZ364751	C1 RF6	PRT	02/08/2019	7397	This study

[a] Country names are indicated with the alpha-3 code (ISO 3166).

Table S2. Relationship between AFP/AFM cases and EV-A71 subgenogroups and Recombinant forms.

**Country**	**Number of cases**	**Age ≥ 2 year**	**Age <2 year**	**Subgenogroup and RFs***
Czech Republic	2	0	2	C1RF9 (**02**)
France	20	13	7	C1 (**14**), C1RF5 (**1**), C2 (**5**)
Hungary	2	2	0	No sequences
Italy	4	0	4	C1 (**3**)
Spain	29	14	15	C1 (**14**), C1RF5 (**13**), C2 (**1**)
**Total**	**57**	**29**	**28**	**53**

*Recombinant forms (RFs) are indicated only when a complete genome sequence was available.

Table S3. Comparison of amino acid substitutions in EV-A71 subclades C1 and C2.

Clade	VP4	VP2	VP3	VP1
C1.1				**I262V, A289T**[#]
C1.2	**A54T** [a]	**V195M**		**M119L**
C1.3		**I170V** [b]**,** V170I*, I219V*	**N93D, A232S** [b]	**V16M***, V262I*, M16V*
C1 (main trunk)	**A54T**	**I170V**, **V195M**	**N93D, A232S**	**V16M**, **M119L, I262V, A289T**
C2 [a]	**T7A,** A7S	S144T*, I170V*, **F224Y**	A62D*, V155I*	**Q22R**, N31D*[c], E145G*[d]

[a] Amino acid changes indicated in bold are located on the main trunk and were used for 3D modelling.
[b] With the exception of MG367595, LR027533.
[c] Multiple occurrences in the tree.
[d] Key position in EV-A71; sequences samples between 1997 & 2001
* Mutations not shared by all sequences (i.e., they are present only on specific branches or lineages).
# amino acids conserved between C1.1, C1.2, C1.3

References

Bankevich, A., Nurk, S., Antipov, D., Gurevich, A.A., Dvorkin, M., Kulikov, A.S., Lesin, V.M., Nikolenko, S.I., Pham, S., Prjibelski, A.D., Pyshkin, A.V., Sirotkin, A.V., Vyahhi, N., Tesler, G., Alekseyev, M.A., Pevzner, P.A., 2012. SPAdes: A New Genome Assembly Algorithm and Its Applications to Single-Cell Sequencing. Journal of Computational Biology 19, 455–477. https://doi.org/10.1089/cmb.2012.0021

Bolger, A.M., Lohse, M., Usadel, B., 2014. Trimmomatic: a flexible trimmer for Illumina sequence data. Bioinformatics 30, 2114–2120. https://doi.org/10.1093/bioinformatics/btu170

Catching, A., Te Yeh, M., Bianco, S., Capponi, S., Andino, R., 2023. A tradeoff between enterovirus A71 particle stability and cell entry. Nat Commun 14, 7450. https://doi.org/10.1038/s41467-023-43029-0

Danecek, P., Bonfield, J.K., Liddle, J., Marshall, J., Ohan, V., Pollard, M.O., Whitwham, A., Keane, T., McCarthy, S.A., Davies, R.M., Li, H., 2021. Twelve years of SAMtools and BCFtools. Gigascience 10, giab008. https://doi.org/10.1093/gigascience/giab008

Kolmogorov, M., Yuan, J., Lin, Y., Pevzner, P.A., 2019. Assembly of long, error-prone reads using repeat graphs. Nat Biotechnol 37, 540–546. https://doi.org/10.1038/s41587-019-0072-8

Li, H., 2013. Aligning sequence reads, clone sequences and assembly contigs with BWA-MEM. https://doi.org/10.48550/arXiv.1303.3997

Lole, K.S., Bollinger, R.C., Paranjape, R.S., Gadkari, D., Kulkarni, S.S., Novak, N.G., Ingersoll, R., Sheppard, H.W., Ray, S.C., 1999. Full-Length Human Immunodeficiency Virus Type 1 Genomes from Subtype C-Infected Seroconverters in India, with Evidence of Intersubtype Recombination. Journal of Virology 73, 152–160. https://doi.org/10.1128/JVI.73.1.152-160.1999

McKenna, A., Hanna, M., Banks, E., Sivachenko, A., Cibulskis, K., Kernytsky, A., Garimella, K., Altshuler, D., Gabriel, S., Daly, M., DePristo, M.A., 2010. The Genome Analysis Toolkit: a MapReduce framework for analyzing next-generation DNA sequencing data. Genome Res 20, 1297–1303. https://doi.org/10.1101/gr.107524.110

Midgley, S.E., Nielsen, A.G., Trebbien, R., Poulsen, M.W., Andersen, P.H., Fischer, T.K., 2017. Co-circulation of multiple subtypes of enterovirus A71 (EV- A71) genotype C, including novel recombinants characterised by use of whole genome sequencing (WGS), Denmark 2016. Euro Surveill 22, 30565. https://doi.org/10.2807/1560-7917.ES.2017.22.26.30565

Nikolaidis, M., Mimouli, K., Kyriakopoulou, Z., Tsimpidis, M., Tsakogiannis, D., Markoulatos, P., Amoutzias, G.D., 2019. Large-scale genomic analysis reveals recurrent patterns of intertypic recombination in human enteroviruses. Virology 526, 72–80. https://doi.org/10.1016/j.virol.2018.10.006

Pettersen, E.F., Goddard, T.D., Huang, C.C., Couch, G.S., Greenblatt, D.M., Meng, E.C., Ferrin, T.E., 2004. UCSF Chimera—A visualization system for exploratory research and analysis. Journal of Computational Chemistry 25, 1605–1612. https://doi.org/10.1002/jcc.20084
